# Lrig1 expression prospectively identifies stem cells in the ventricular-subventricular zone that are neurogenic throughout adult life

**DOI:** 10.1186/s13064-020-00139-5

**Published:** 2020-03-17

**Authors:** Hyung-song Nam, Mario R. Capecchi

**Affiliations:** Department of Human Genetics, University of Utah School of Medicine, Salt Lake City, UT 84112-5331 USA

**Keywords:** Lrig1, Stem cells, Adult neurogenesis, Ventricular-subventricular zone, Single cell RNA sequencing, Genetic inducible fate mapping, Inbred mice, C57BL/6J

## Abstract

**Background:**

*Leucine-rich repeats and immunoglobulin-like domains 1* (*Lrig1*) regulates stem cell quiescence. As a marker, it identifies stem cells in multiple organs of the mouse. We had detected *Lrig1* expression in cultured Id1^high^ neural stem cells obtained from the lateral walls lining the lateral ventricles of the adult mouse brain. Thus, we investigated whether Lrig1 expression also identifies stem cells in that region in vivo.

**Methods:**

Publicly available single cell RNA sequencing datasets were analyzed with Seurat and Monocle. The Lrig1+ cells were lineage traced in vivo with a novel non-disruptive co-translational *Lrig1*^*T2A-iCreERT2*^ reporter mouse line.

**Results:**

Analysis of single cell RNA sequencing datasets suggested *Lrig1* was highly expressed in the most primitive stem cells of the neurogenic lineage in the lateral wall of the adult mouse brain. In support of their neurogenic stem cell identity, cell cycle entry was only observed in two morphologically distinguishable Lrig1+ cells that could also be induced into activation by Ara-C infusion. The Lrig1+ neurogenic stem cells were observed throughout the lateral wall. Neuroblasts and neurons were lineage traced from Lrig1+ neurogenic stem cells at 1 year after labeling.

**Conclusions:**

We identified Lrig1 as a marker of long-term neurogenic stem cells in the lateral wall of the mouse brain. Lrig1 expression revealed two morphotypes of the Lrig1+ cells that function as long-term neurogenic stem cells. The spatial distribution of the Lrig1+ neurogenic stem cells suggested all subtypes of the adult neurogenic stem cells were labeled.

## Background

Adult stem cells retain the capacity to self-renew and the potential to differentiate. The adult stem cells of the mammalian brain – adult neural stem cells – are astrocyte-like cells, and are observed in at least two major niches, the ventricular-subventricular zone (V-SVZ) near the lateral ventricles and the subgranular zone (SGZ) in the hippocampal dentate gyrus (recently reviewed in [1]).

In mice, generation of newborn neurons in the brain through adult life (adult neurogenesis) persists in the neurogenic niches, where the adult neural stem cells sustain this process [2, 3]. At each cell division that ultimately gives rise to new neurons, the neural stem cell pool is maintained by asymmetric or symmetric self-renewal of the constituent stem cells [4]. Self-renewal of the stem cells maintains the pool because at least one progeny cell retains its “stemness” after cell division. However, it is becoming apparent that the self-renewal capacity may not be infinite for adult neural stem cells, perhaps due to genetic limits to cell replication [5]. After several cell divisions, the adult neural stem cells die, are consumed, or terminally differentiate into astrocytes [6–9]. For these reasons, some adult neural stem cells are postulated to maintain a life-long stem cell pool by remaining out of the cell cycle altogether, in dormancy or quiescence [10–13].

To study the adult neural stem cells in vivo, we searched for a marker gene that can prospectively identify these cells and label them robustly in mice. We identified a candidate gene *Lrig1* (*Leucine-rich repeats and immunoglobulin-like domains 1* [14]) from our previous work [15]. Lrig1 maintains quiescence by negatively regulating mitogenic signals from receptors such as the epidermal growth factor receptor (EGFR, reviewed in [16]). *Lrig1* regulates quiescence of cultured skin stem cells [17]. *Lrig1* was recently utilized as an in vivo stem cell marker in the intestine and the skin [18, 19]. We hypothesized that Lrig1 expression could also prospectively identify quiescent stem cells in the brain because EGF – the ligand of the EGFR that Lrig1 down-regulates – is potently mitogenic for the EGFR-expressing activated neural stem cells [2, 12, 20].

In this study, we investigated the Lrig1+ adult stem cells in the V-SVZ stem cell niche in the lateral wall lining the lateral ventricles using multiple approaches. The V-SVZ stem cells were studied because the ventricular wall whole mount technique [21] enabled single cell resolution histological analysis of the entire V-SVZ niche. First, consistent with our hypothesis, a bioinformatic analysis of single cell RNA sequencing datasets in the public domain [13, 22, 23] suggested that *Lrig1* is indeed expressed in stem cells of the V-SVZ neurogenic lineage. Second, with a novel knock-in mouse line, we observed the generation of reporter-labeled neuroblasts and neurons throughout adult life, indicating that the Lrig1+ stem cells are neurogenic in vivo. Third, by analyzing the cell cycle entry of the various Lrig1+ cells in the V-SVZ at steady-state and after injury, we could implicate a morphologically distinguishable subset of all Lrig1+ cells as the stem cells of the neurogenic lineage. Thus, we have identified Lrig1+ neurogenic stem cells in the lateral wall that generate olfactory bulb interneurons throughout adult life.

## Methods

### Bioinformatics

Scripts were written in the R language environment [24] to analyze the single cell RNA sequencing datasets obtained from the NCBI database. The counts files were read into R and formatted. Using Seurat (version 2.3.4), cells were filtered based on number of genes and percent of mitochondria reads after graphical determination of the cut-off parameters. Following normalization, the counts were regressed for the number of UMI’s, mitochondria reads, and cell cycle genes. The regressed counts were scaled. Variable genes were then scored with the scaled data. Then, PCA and UMAP dimensional reductions were performed on the data. Finally, clusters were identified with the resolution setting at 0.8 and 1 to 20 dimensions. Marker genes and differentially expressed gene lists were determined from the clustering. For pseudotime ordering, using Monocle (version 2.12.0), cells determined to be in the neurogenic lineage by the clustering were imported and analyzed. After estimation of size factors and dispersions, expressed genes were detected. A cell type hierarchy was set up for semi-supervised clustering. The cells were classified, and differential markers were identified. Dimensional reduction was applied, then the cells were ordered. To validate the results of clustering and pseudotime ordering, the clusters and ordering were compared against the known biology of the V-SVZ cells in [23, 25].

### Mouse experimentation

Mice were housed in Thoren rack cages. After weaning at 3–4 weeks, 3–4 same-sex littermates were housed in each cage with bedding and paper nestlets as environmental enrichment. Mice were transferred to fresh cages every 2 weeks. Food and water were provided ad libitum. The food was a custom formulation referred to as the Capecchi diet.

### Mouse genetics

The novel alleles introduced in this work, *Lrig1*^*T2A-iCreERT2*^, *Lrig1*^*T2A-tdTomato*^, and *Cdk6*^*T2A-td-sfGFP*^, were generated by conventional gene targeting procedure. Targeting vectors were constructed with homology arms subcloned from the RPCI-24 library of C57BL/6J mouse genomic DNA (CHORI). The vectors were electroporated into the G4 embryonic stem cells derived from 129S6/SvEvTac × C57BL/6Ncr F1 embryos [26]. The resulting germline mice were bred to *Actb-FLPe* or *Pgk1-FLPo* transgenic mice [27, 28] obtained from The Jackson Laboratory. Because the C57BL/6N mouse genome harbors a retinal degeneration mutation, *rd8* [29], that may affect behavioral experiments, the mice were backcrossed to the C57BL/6J background while fixing the X and Y chromosomes to that background (the C57BL/6J mouse stock from The Jackson Laboratory). At the time of manuscript preparation, the three lines had been backcrossed for more than 8 generations to the C57BL/6J background.

The *ROSA26*^*Ai14*^ mouse line [30] obtained from the Allen Institute for Brain Science was backcrossed to the C57BL/6J background for 7 generations by Simon Titen, Capecchi laboratory.

The Coffey *Lrig1*^*creERT2*^ mouse line [19] obtained from The Jackson Laboratory was backcrossed to the C57BL/6J background for 4 generations. The Coffey *Lrig1* allele is a null allele because *creER*^*T2*^ cDNA completely replaces the first exon coding sequence which encodes Lrig1’s signal sequence to the plasma membrane.

The *Lrig1*^*T2A-iCreERT2/+*^*; ROSA26*^*Ai14/+*^ mice were bred to be genetically as similar as practically possible to each other: *Lrig1*^*T2A-iCreERT2/+*^ or *Lrig1*^*T2A-iCreERT2/T2A-iCreERT2*^ brothers were bred to *ROSA26*^*Ai14/Ai14*^ sisters. Thus, the mice in experimental cohorts were not systematically randomized. Both sexes were included in the cohorts.

The targeting vectors and the mouse strains will be available from Addgene and The Jackson Laboratory.

### Assay for the SNP near *Lrig1* 3′ UTR

Wildtype embryonic stem cells and targeted embryonic stem cell clones were adapted to feeder-free culture condition on gelatinized plates in 2i media [31]. Genomic DNA was purified from the feeder-free cultures. A 3′ region of *Lrig1* was amplified by PCR with Taq (Takara). The PCR cycling program was 95 °C 3 min, (95 °C 20 s, 60 °C 30 s, 72 °C 2 min) × 30 cycles, 72 °C 2 min. The PCR products were purified, then digested with BstZ17I (NEB). The PCR primers were:

*Lrig1* targeted clone genotyping

5’-GCCAGAGGCCACTTGTGTAG-3’

5’-GACCCATGCGCTAAGGATTA-3’

5’-TCCAAGCAACCATGACAGAA-3’

Wildtype allele 692 bp, knock-in allele 477 bp

*Lrig1* 3’ SNP genotyping

5’-CTGTTGTCCCGACAAGGTTT-3’

5’-AGCCGACTGACTGACATTCC-3’

1392 bp

### Mouse genotyping

Ear clips from the mice were processed by HotSHOT method [32], then genotyped by PCR with Taq (Takara). The PCR cycling program was 95 °C 3 min, (95 °C 20 s, 60 °C 30 s, 72 °C 1 min) × 30 cycles, 72 °C 2 min. The genotyping PCR primers were:

*Lrig1*^*T2A-iCreERT2/+*^ and *Lrig1*^*T2A-tdTomato/+*^ mouse

5’-AGCTCATGGAAGACGCCATA-3’

5’-CCAGATGCCACTCCTCTAGC-3’

5’-CCGGATCCATTATGTACCTGAC-3’

302 bp wildtype allele, 211 bp knock-in allele

Coffey *Lrig1*^*creERT2/+*^ mouse

5’-GACTCGCTGGACTGCAGT-3’

5’-CCGTCTCACATGCACACAAA-3’

5’-CGAGTGATGAGGTTCGCAAG-3’

5’-TTCACCGGCATCAACGTTTT-3’

483 bp wildtype allele, 332 bp cre cDNA

*Cdk6*^*T2A-td-sfGFP/+*^ mouse

5’-AGGGCAGCTTGAAGAAAGGT-3’

5’-CCGGATCCATTATGTACCTGAC-3’

5’-TGCAGGCGGATTACATCATA-3’

785 bp wildtype allele, 456 bp knock-in allele

*ROSA26*^*Ai14/+*^ mouse

5’-TTATGTAACGCGGAACTCCA-3’

5’-GCACTTGCTCTCCCAAAGTC-3’

5’-GGCGGATCACAAGCAATAAT-3’

445 bp wildtype allele, 320 bp knock-in allele

To genotype the *rd8* mutation [29], PCR cycling program was 94 °C 5 min, (94 °C 30 sec, 65 °C 30 sec, 72 °C 1 min) × 30 cycles, 72 °C 7 min. The primers were:

5’-GCCCCATTTGCACACTGATGAC-3’

5’-GCCCCTGTTTGCATGGAGGAAACTTGGAAGACAGCTACAGTTCTTCTG-3’

244 bp

### 2A-creER variants test

A cell-based assay was performed with the NIH/3T3 transformed mouse fibroblasts (ATCC) and plasmids. Plasmids were constructed using standard methods and purified with midiprep columns (Machery-Nagel). All purified plasmids were quantitated with a spectrophotometer in one session. 2.5 × 10^5^ cells were seeded to each well of a 6-well plate. Following day, the cells were transfected in triplicates with molar equivalents of plasmids (i.e., 0.5–0.55 μg of creER, 0.5 μg of reporter, and 1 μg of pBluescript II SK- carrier) using 3-fold jetPRIME transfection reagent (Polyplus-transfection). Next day, media was changed, and creER was induced by addition of 4-hydroxytamoxifen (Sigma) dissolved in ethanol to the media at 10 nM. Next day, the cells were detached with Accutase (Gibco), and resuspended in HBSS (Gibco) with DRAQ7 (Biostatus). The cell suspensions were analyzed on a custom FACSCanto flow cytometer (BD). Single live cells were gated for BFP fluorescence. The percentages of RFP+ cells among these cells were determined for each sample.

### Mouse home cage behavior measurement with the Laboras platform

Laboras platform (Metris) was set up with one mouse per cage and run per manufacturer’s instructions. Home cage behaviors were measured from 10 to 11 am for 24–48 h.

### Tamoxifen induction

Tamoxifen (Sigma) was solubilized in 90% corn oil (Sigma) and 10% ethanol vehicle. Fresh tamoxifen formulation was prepared about an hour before injection by warming the suspension at 37 °C and solubilizing with a Branson sonifier and vortexer. Thorough sonification was critically necessary for efficient induction. Every mouse in a cohort was weighed, then intraperitoneally injected a calculated volume of the tamoxifen formulation once. All mice in the cohort were injected in one session and returned to fresh cages. The cages were changed again at 3 days after the injection. Inductions were more variable with higher injection volumes, and were most reproducible at volumes of 50–100 μl. Thus, we injected 20 mg/ml stock to 40 mg/kg, 40 mg/ml stock to 80 mg/kg, 60 mg/ml stock to 230 mg/kg, and so on. Inductions of male and female mice were comparable because the injection volumes were adjusted for the mouse weight differences.

### X-gal histochemistry

X-gal staining was performed as in [15].

### Flow cytometric analysis of dissociated V-SVZ cells

Lateral walls dissected from the brains in ice-cold HBSS (Gibco) were dissociated to single cells with papain and DNase I (Worthington) in EBSS (Gibco). Papain was titrated down to minimize Lrig1 proteolysis. Then, the cells in the dissociate was purified from the debris by filtration and centrifugations through solutions of sucrose and BSA (Sigma) [33]. The resulting single cell suspensions were stained with antibodies and washed in 0.5% BSA (Jackson ImmunoResearch) in PBS. Stained cells were resuspended in HBSS with DAPI (Sigma), then analyzed with a custom FACSCanto flow cytometer (BD) with a 488 nm laser for the td-sfGFP and a 561 nm laser for the tdTomato.

### Thymidine analog administration

EdU (ethynyl deoxyuridine, Carbosynth) was administered in drinking water for 7 days at 0.15 mg/ml with 1% glucose to avoid taste aversion. In preliminary experiments, the EdU dose was titrated down. The low dose was minimally toxic, and did not reduce the number of Ascl1+, Ki-67+, or Dcx+ cells in the lateral wall as determined by whole mount immunofluorescence analyses. Higher EdU doses, e.g., 0.8 mg/ml, did.

### Ara-C infusion

Ara-C (Sigma) dissolved to 2% in artificial cerebrospinal fluid (Tocris) was infused for 6 days into one of the two lateral ventricles with an osmotic minipump (Alzet) as in [15].

### Whole mount immunofluorescence

Mice were transcardially perfused with room-temperature PBS with 20 U/ml heparin then ice-cold 2% PLP fixative composed of 2% formaldehyde, 75 mM lysine, 10 mM NaIO_4_, and 0.1 M phosphate buffer pH 7.4 [34]. Formaldehyde dissolved in water from paraformaldehyde powder was mixed with lysine and sodium periodate dissolved in phosphate buffer before perfusions. Perfused brains were dissected out, rinsed in PBS, then bisected and further dissected in PBS to reveal the lateral wall [21]. The dissected brains were post-fixed overnight in 2% PLP at 4 °C on a nutator. 2% PLP did not over-fix the specimen, whereas 4% PLP presumably did as evident by drop in immunoreactivity to some antibodies (e.g., the rat anti-Ki-67 antibody). The brains were well- and not under-fixed, as they were stable and stained well even after a year in refrigeration. The fixed brains were rinsed in PBS, then blocked with 0.3 M glycine in PBS pH 7.4 overnight at 4 °C on nutator. The brains were trimmed in PBS, and then permeabilized in 0.5% Triton X-100 in PBS at room temperature. The brains were blocked with 10% normal goat serum (Vector Labs), 20 μg/ml goat anti-mouse IgG F(ab) fragment (Jackson ImmunoResearch), 0.5% BSA (Jackson ImmunoResearch), and 0.1% TX-100 in PBS at 4 °C on nutator. After brief washes in PBS + 0.1% TX-100 (PBST) at room temperature, the brains were nutated for 48 h with primary antibodies in 1% normal goat serum, 0.5% BSA, and 0.1% TX-100 in PBS at 4 °C. After four washes in PBST at room temperature, the brains were again nutated with cross-adsorbed DyLight 405, Alexa 488, Rhodamine Red-X, Alexa 555, and/or Alexa 647 secondary antibodies (Jackson ImmunoResearch, Invitrogen) as before for 24 h. The brains were washed 3 times in PBST then 1 time in TBST (100 mM Tris pH 8.5, 150 mM NaCl, 0.1% TX-100) at room temperature. In early experiments that did not involve density or area measurements, a slice of the lateral wall was cut freehand with a scalpel. In later experiments, a slice was cut with a custom 3D printed jig and a razor. The slice was trimmed in TBS (minus TX-100) then coverslipped with a #1.5 cover glass (Fisher Scientific) within a 0.5 mm spacer (Invitrogen) in a mounting media composed of 10% w/v Mowiol (Polysciences), 25% w/v glycerol (Sigma), and 100 mM Tris pH 8.5.

### EdU detection on whole mounts

Click chemistry for EdU detection [35] was performed after permeabilization and before the antibody staining with Alexa 488-picolyl azide or Alexa 647-picolyl azide (Invitrogen, Jena Bioscience). To reduce the background from copper sulfate, the brains were washed with distilled water before and after click chemistry.

### Immunofluorescence on sections

Brains fixed and blocked as described above were cryo-protected in solutions of sucrose in PBS of increasing concentrations, then frozen in OCT (Sakura). Fifty-micron sections were cut on a cryostat (Leica). Antibody staining was performed with the same buffers as for the whole mounts, then coverslipped with Mowiol.

### Primary antibodies

Ascl1 (Mash1) mouse monoclonal clone 24B72D11.1 BD Biosciences 1:200

β-catenin mouse monoclonal clone 14/Beta-catenin BD Biosciences 1:500

Dcx guinea pig polyclonal Milipore 1:5000

Dcx rabbit polyclonal Cell Signaling Tech 1:500

Gfap mouse monoclonal clone GA5 Cell Signaling Tech 1:100

Gfap rat monoclonal clone 2.2B10 Invitrogen 1:100

GFP chicken polyclonal Rockland Immunochemicals 1:500

γ-tubulin mouse monoclonal clone GTU-88 Sigma 1:500

Ki-67 rat monoclonal clone SolA15 Invitrogen 1:200

Lrig1 goat polyclonal R & D Systems 1:100

Mcm2 mouse monoclonal clone 2B3 Sigma 1:100

NeuN mouse monoclonal clone A60 Millipore 1:1000

Pdgfra rabbit monoclonal clone D1E1E Cell Signaling Tech 1:100

RFP guinea pig polyclonal Frontier Institute 1:500

RFP rabbit polyclonal Rockland Immunochemicals 1:500

RFP rat monoclonal clone 5F8 Chromotek 1:100

S100 rabbit polyclonal Invitrogen 1:500

Sox2 mouse monoclonal clone 14A6A34 BioLegend 1:100

### Confocal imaging

Images were acquired with a confocal microscope (Leica) and analyzed with FIJI [36].

### Cell counting methods

#### Counting the RFP+ cells in the lateral walls

A cell counting workflow using FIJI and R was established. An R package for spatial statistics, spatstat [37], was utilized. Because the whole mount confocal images were not cropped, two sets of coordinates were generated to count the RFP+ cells only in the region of interest. First set of coordinates included manually clicked borders of the region of interest. Second set of coordinates included locations of all RFP+ cells. This was scored manually in FIJI. The coordinates were input into R, then the number of RFP+ cells within the region of interest was counted by the “nobjects” function.

#### Measuring the Dcx+ area in the lateral wall

From the Dcx immunofluorescence image z-stack, background was subtracted with “Subtract Background.” A maximum projection was generated with “Z Project.” Levels between the images were adjusted to equivalence with “Enhance Contrast” and applied in “Window/Level.” After thresholding with “Threshold”, thresholded area was measured by “Measure.” This Dcx+ area was divided by the total area of the lateral wall measured in FIJI.

#### Counting the RFP+ cells in proliferating clusters and the RFP+ Dcx+ neuroblasts

After imaging the entire whole mounts with the four antibodies combination, the slides were taken apart, and the whole mounts were stained with DAPI before being coverslipped again. Then, each cluster of proliferating cells was imaged again as a z-stack at 40x on the confocal microscope. To ease the visualization of the co-localizing signals, the following image processing steps were taken. With the z-stacks, co-localized RFP and DAPI signals were determined using the “Colocalization Threshold” function to create silhouettes of the nuclear RFP signal. The silhouettes of nuclear RFP signal thus generated were overlaid back to the DAPI signal that better revealed each distinct nucleus. Visually inspecting the z-stack of the processed nuclear RFP signal and DAPI signal, one distinct nucleus with overlaid RFP signal was scored as a single cell.

RFP+ neuroblasts were counted after similar processing steps to highlight the Dcx+ RFP+ cells among all RFP+ cells in the lateral wall. RFP+ Dcx signal among all Dcx signal was thresholded as above. Then, the thresholded RFP+ Dcx signal was overlaid with the RFP signal. The Dcx+ RFP+ neuroblasts thus identified were scored in FIJI and counted using the R workflow described above.

#### Counting RFP+ olfactory bulb interneurons

To count the RFP+ interneurons in the olfactory bulb, olfactory bulb coronal sections were imaged in entirety and stitched. With these images, each RFP+ interneuron was scored in FIJI. The borders of the bulb and granular cell layer were defined in FIJI. The three zones of the granular cell layer were calculated by an R script to be three one-thirds of the total granular cell layer area. The RFP+ interneurons in each zone were counted by R.

#### Counting Ascl1+ or Ki-67+ nuclei in a region of interest

To count Ascl1+ or Ki-67+ nuclei in a square region of interest, “Spots” function in Imaris was utilized on z-stacks.

### Statistics

Statistical significance was calculated in R with scripts using “shapiro.test,” “t.test,” “wilcox.test,” “aov,” “kruskal.test,” “pairwise.t.test,” and “pairwise.wilcox.test” as appropriate. Briefly, normality was determined with Shapiro-Wilk test. For pairwise comparisons, Student’s t test or Mann-Whitney U test were performed depending on the normality of the samples. For multiple comparisons, differences in the samples were determined with ANOVA or Kruskal-Wallis test depending on the normality of the samples. Post-hoc tests were performed with pairwise Student’s t test or pairwise Mann-Whitney U test.

## Results

### *Lrig1* mRNA identifies stem cells in the adult V-SVZ neurogenic lineage

In addition to our line of investigation (Additional file 1), others have also previously observed *Lrig1* expression in the V-SVZ quiescent neural stem cells (qNSC’s) [12]. To corroborate these observations and to determine whether *Lrig1* would be a useful marker for further investigations, we analyzed the single cell RNA sequencing datasets in the public domain [13, 22, 23]. Bioinformatic analysis of the three datasets with established software programs [38, 39] yielded similar results. Here, we present our analysis of the largest dataset from [23].

**Fig. 1:**
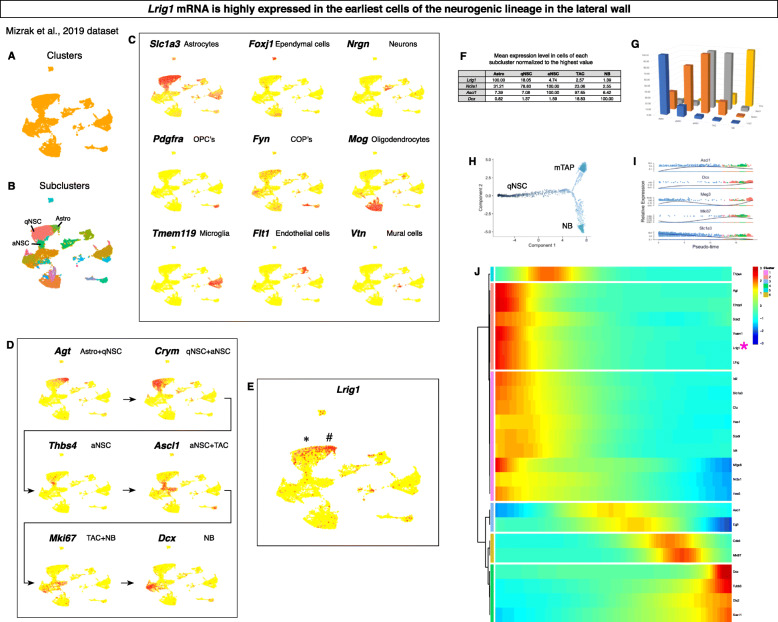
*Lrig1* as a quiescent neurogenic stem cell marker. **a** Clusters of different cell types identified from single cell RNA sequencing dataset in [23]. **b** Subclusters identified within clusters of different cell types. **c** Identification of each cluster’s identity by canonical marker genes. **d** Progression of neurogenic markers within two linked clusters. **e** Expression pattern of *Lrig1*. **f** Mean of marker gene expression levels in cells of each subcluster. **g** Graph of the expression levels. **h** Pseudotime ordering of the neurogenic lineage cells. **i** Trends of known transcripts for validation. **j** Pseudotime heat map of canonical marker gene transcripts

Using the Seurat program, we first clustered the cells in an unbiased way. This step yielded distinct clusters (Fig. 1a) as well as subclusters within some clusters (Fig. 1b). The identity of the cells within each cluster was revealed by plotting the expression levels of known marker genes from [23] (Fig. 1c). Then, we confirmed the neurogenic lineage progression within two clusters by plotting additional known marker genes (Fig. 1d). For example, *Agt* expression was highest in the astrocyte cluster then high in the qNSC cluster. *Lrig1* was expressed in a pattern very similar to *Agt* (Fig. 1e).

To determine in what subclusters *Lrig1* expression is the highest, we calculated the mean expression level within each subcluster. To verify the accuracy of the result, several well-known marker genes were also analyzed: *Nr2e1* [40, 41], *Ascl1* [42], and *Dcx*. As expected, this analysis revealed that *Nr2e1* and *Ascl1* expression levels peaked in activated stem cells, and *Dcx* expression level peaked in neuroblasts (Fig. 1f-g). In contrast, *Lrig1* expression level was highest in the astrocyte subcluster, high in the qNSC then steeply decreased along the neurogenic lineage.

To extend this analysis, we constructed a “pseudotime” lineage of the neurogenic lineage cells using the Monocle program (Fig. 1h). The accuracy of the ordering was confirmed by comparison to the ordering in [25] (Fig. 1i). The gene expression trends in the two pseudotime lineages were identical. With the ordered cells, we generated a pseudotime gene expression heatmap. In this analysis, *Lrig1* transcript levels were the highest in the cells ordered first in the pseudotime (i.e., in the astrocytes) and remained high (i.e., in the qNSC’s) until the *Thbs4*+ activated stem cells (Fig. 1j). This was consistent with the trend obtained from the manual calculation of the expression levels in the neurogenic lineage subclusters. Taken together, these analyses suggested that the stem cells at the earliest steps of neurogenic differentiation express high levels of *Lrig1*.

### *Lrig1*^*T2A-iCreERT2*^ knock-in allele

**Fig. 2:**
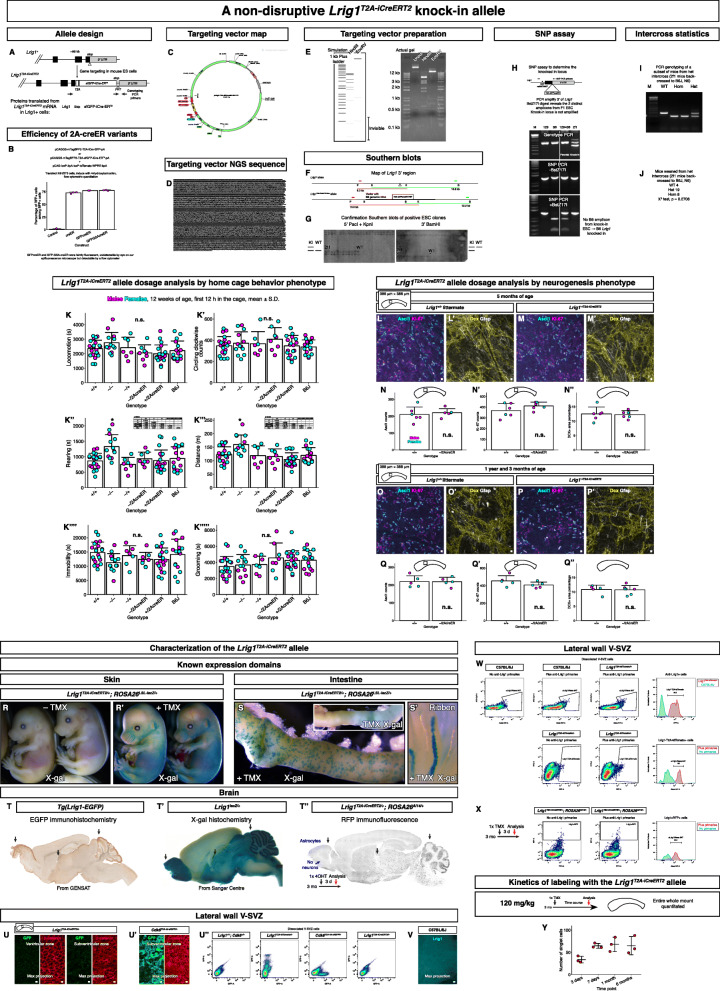
A non-disruptive *Lrig1*^*T2A-iCreERT2*^ reporter allele. **a** Allele design. **b** Comparison of recombinase variants efficiencies in a cell-based recombination assay. Mean ± standard deviation. **c** Targeting vector map. **d** Next generation sequencing sequence of the entire targeting vector. **e** Restriction digests of the targeting vector prepared for the electroporation. **f** Southern blot strategy. P, PacI. B, BamHI. K, KpnI. **g** Confirmation Southern blots. **h** SNP analysis to determine the knocked in allele. **i** Genotyping results of a subset of mice from heterozygote × heterozygote intercross. **j** Results of a Chi-square statistic calculation from intercross litters. **k**-**k’’’’’** Summary of several behavioral parameters measured by Laboras. Asterisk, significant sample. N.s., not significant. Mean ± standard deviation. **l**-**m’** Neurogenic markers expression in young mice. Scale bar, 10 μm. **n**-**n”** Comparison of Ascl1 and Ki-67 counts in young mice as well as Dcx+ pixel areas. Mean ± standard deviation. **o**-**p’** Neurogenic markers expression in old mice. Scale bar, 10 μm. **q**-**q”** Comparison of Ascl1 and Ki-67 counts in old mice as well as Dcx+ pixel areas. Mean ± standard deviation. **r**-**r’** Recombinase activity in the skin when induced with tamoxifen. **s**-**s’** Recombinase activity in the intestine when induced with tamoxifen. A ribbon from the intestinal crypt is shown. **t**-**t”** Recombinase activity in the brain matches other *Lrig1* reporter alleles. Expression is observed in the olfactory bulb, midbrain, and cerebellum. **u**-**u’** The sfGFP-iCre-ER^T2^ from the allele cannot be detected by indirect immunofluorescence. Scale bar, 10 μm. **u”** The sfGFP-iCre-ER^T2^ from the allele cannot be detected by flow cytometry even though *Lrig1* is expressed in the V-SVZ. **v** The Lrig1 protein also cannot be detected in the V-SVZ by indirect immunofluorescence. Scale bar, 10 μm. **w**-**x** However, the Lrig1 protein can be detected by flow cytometry, and is expressed in nearly all of the RFP+ cells labeled by the *Lrig1*^*T2A-iCreERT2*^ allele. **y** Numbers of singlet RFP+ cells demonstrate the kinetics of labeling with the allele. Mean ± standard deviation

The analysis above suggested *Lrig1* could be a useful marker gene with which to analyze the V-SVZ neurogenic stem cells in vivo. Mouse lines were generated to determine the effectiveness of *Lrig1* as a genetic marker gene. Previously, two “knock-in knock-out” transcriptional reporter lines of *Lrig1* were generated [18, 19]. There is no haploinsufficiency phenotype because of *Lrig1* heterozygosity (also see below). Nevertheless, we generated a non-disruptive co-translational reporter allele using the 2A ribosome skip sequence [43] because this design allows multiple reporter alleles [44], even of the same gene. A *T2A-sfGFP-iCre-ER*^*T2*^*-FNF* cassette was knocked in between the end of the coding sequence and the 3′ untranslated region while removing the endogenous stop codon (Fig. 2a). Utilizing a similar design, an *Lrig1*^*T2A-tdTomato*^ allele and a *Cdk6*^*T2A-td-sfGFP*^ allele were also generated.

To characterize the sfGFP-iCre-ER^T2^ recombinase protein we utilized, cell-based assays were performed with the NIH/3T3 transformed mouse fibroblasts. First, as expected, the linker between sfGFP and iCre-ER^T2^ had no ribosome skip activity. TdTomato fused to H2B-sfGFP with that linker localized to the nucleus [45] (data not shown). Second, the fusion of the iCre-ER^T2^ fragment to sfGFP with that linker almost completely abolished the sfGFP fluorescence in the cells (data not shown). Replacing the linker with a synthetic (Glycine-Serine-Alanine) × 9 linker did not restore the sfGFP fluorescence (data not shown). This suggested the iCre-ER^T2^ rather than the linker was destabilizing. Third, comparing the 4-hydroxytamoxifen induced cre activity revealed no difference in activities between the three variants (Fig. 2b). Thus, the expression of the sfGFP-iCre-ER^T2^ fusion protein from the CAG promoter resulted in faint sfGFP fluorescence in the cells, possibly due to short half-life of ER^T2^ [46, 47] and/or iCre in the cell, but its tamoxifen-inducible recombinase activity was identical to the activity of iCre-ER^T2^.

The targeting vector (Fig. 2c) was constructed. Next generation sequencing of the entire targeting vector confirmed the vector sequence (Fig. 2d). To transmit the *Lrig1*^*T2A-iCreERT2*^ allele through the germline, we utilized the G4 129 × B6 F1 hybrid embryonic stem cells [26] in which one of the two sets of autosomes are of the B6 background. Southern blot hybridizations of the ES cells’ genomic DNA (Fig. 2f) revealed many positive clones with *Lrig1* 3′ region targeted in one of the two chromosomes (Fig. 2g). Importantly, a SNP assay [48] revealed that the B6 *Lrig1*^*T2A-iCreERT2*^ targeting vector recombined into *Lrig1* on a B6 chromosome (Fig. 2h). The mice resulting from these ES cells were backcrossed to the C57BL/6J background for six generations. When heterozygotes of this C57BL/6J congenic mouse line were interbred, normal homozygotes could be weaned at Mendelian ratios (Fig. 2i-j). Furthermore, these homozygotes could breed normally with each other and generate normal size litters (data not shown), suggesting that the homozygosity of this allele does not result in adverse developmental effects even in the inbred background. This result also confirmed that *Lrig1* must have been knocked in initially and carried through the generations.

### *Lrig1*^*T2A-iCreERT2*^ knock-in allele is non-disruptive

We sought to establish that our *Lrig1* knock-in allele is not a hypomorph. The Lrig1 protein is processed into multiple fragments making it difficult to quantify by Western blots. Thus, the dosage analysis paradigm from classical genetics was utilized. Because *Lrig1* heterozygous mice are normal (see below), we could generate trans-heterozygous mice, i.e., mice with the *Lrig1*^*T2A-iCreERT2*^ allele over a null allele (the Coffey *Lrig1*^*creERT2*^ allele [19]). If the *Lrig1*^*T2A-iCreERT2*^ allele is normal, the reduction in the *Lrig1* dosage from the null allele is “complemented” by this allele, and no phenotypes should be evident. As for the phenotypes to quantify, a behavioral and a cellular phenotype were sought in the *Lrig1* knock-out mice.

Mouse behaviors were measured in an unbiased way with the commercial Laboras platform (https://www.metris.nl/en/products/laboras/). Measurement over 3 days suggested that the platform could measure changes in mouse home cage behaviors (data not shown). Essentially, after the mice are put into the Laboras platform cage, the mice explore the new cage environment over the first 12 h, then are acclimated in the next day and a half. At that point, the mice can be stimulated by caffeine administration in drinking water (data not shown). In this paradigm, the *Lrig1* knock-out mice showed statistically significant differences during the first 12 h in some of the measures related to exploratory behavior. Increases in the distance traveled and rearing (i.e., standing up on hindlimbs and looking around at the edge of the cage) were observed (Fig. 2k’’-k’’’). Next, we measured cellular phenotypes in the lateral wall using protein markers of stem cell proliferation and neurogenesis. Preliminary experiments suggested that our immunostaining protocol revealed all Ki-67+, all Mcm2+, all Ascl1+, and all Dcx+ cells in the lateral wall (Additional file 2). *Lrig1* knock-out mice showed increased proliferation in the V-SVZ (data not shown, a manuscript in preparation) consistent with the previous reports on the intestinal crypt stem cell niche [19, 49].

Importantly, the increases in the distance traveled and rearing were absent in the *Lrig1* heterozygous mice and the trans-heterozygous mice. Furthermore, the counts of Ascl1+ nuclei and Ki-67+ nuclei as well as Dcx+ pixel areas in the trans-heterozygous mice were comparable to wildtype mice when young and old (Fig. 2l-q”). These indicated that the *Lrig1*^*T2A-iCreERT2*^ allele must lead to production of functional Lrig1 protein because the trans-heterozygous mice don’t show the behavioral and cellular phenotypes observed in the knock-out mice. Thus, we infer that our knock-in *Lrig1*^*T2A-iCreERT2*^ allele is not a hypomorph.

**Fig. 3:**
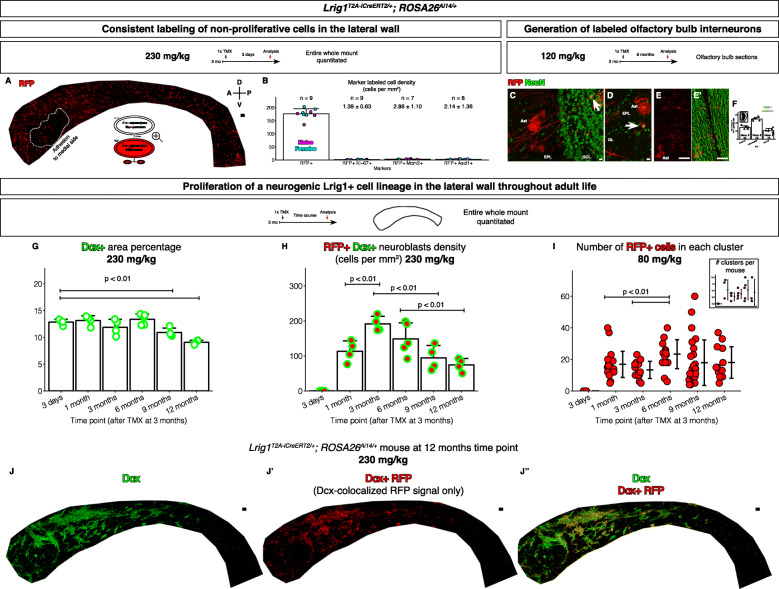
An Lrig1+ cell lineage remains neurogenic throughout adult life. **a** *Lrig1*^*T2A-iCreERT2/+*^; *ROSA26*^*Ai14/+*^ mice induced with a single injection of 230 mg/kg tamoxifen (TMX). Lateral walls visualized at day 3 by whole mount immunofluorescence and confocal microscopy. Scale bars, 100 μm. **b** Reproducible induction efficiency. Mostly non-proliferative cells were labeled. Ki-67+ RFP+ cells, Mcm2+ RFP+ cells, and Ascl1+ RFP+ cells were very rare. Each *n* is one lateral wall from one mouse. Mean ± standard deviation. **c** An RFP+ NeuN+ granule cell in the olfactory bulb. Scale bar, 10 μm. **d** An RFP+ NeuN+ periglomerular cell in the olfactory bulb. Scale bar, 10 μm. **e**-**e’** Zoomed out image of the olfactory bulb showing numerous RFP+ NeuN+ cells with the morphology of interneurons. Scale bar, 100 μm. **f** Counts of RFP+ NeuN+ interneurons from serial sections of an olfactory bulb from a mouse demonstrating that more than one hundred RFP+ interneurons were labeled per 50 μm section of the olfactory bulb. Mean ± standard deviation. **g** Dcx+ pixel area in the lateral wall over time. Mean ± standard deviation. **h** Dcx+ RFP+ cells density (cells/mm^2^) in the lateral wall over time. Mean ± standard deviation. **i** Numbers of RFP+ cells in proliferating clusters over time. Mean ± standard deviation. Inset, total number of clusters per mouse. Mean ± standard deviation. **j**-**j”** An experimental mouse at 1 year after tamoxifen induction. **j** Dcx immunoreactivity. Scale bars, 100 μm. **j’** Dcx-co-localized RFP immunoreactivity. Scale bars, 100 μm. **j”** Extent of the RFP+ Dcx immunoreactivity, i.e., readout of the neurogenesis from the Lrig1+ cell lineage. Scale bars, 100 μm

We also compared the expression pattern of Dcx through aging in wildtype and trans-heterozygous mice uninjected with tamoxifen and *Lrig1*^*T2A-iCreERT2/+*^; *ROSA26*^*Ai14/*+^ mice injected tamoxifen once (Fig. 2q” and Fig. 3g). There were no measurable differences. Taken together, these analyses established that the mouse line that carries the non-disruptive *Lrig1*^*T2A-iCreERT2*^ allele is phenotypically indistinguishable from C57BL/6J mice in the measurements we performed when young and old.

### Characterization of the *Lrig1*^*T2A-iCreERT2*^ allele

*Lrig1* is expressed in the skin and the intestinal crypt stem cell niche, as well as in other organs [18, 19, 50]. Tamoxifen induction in developing embryos and in adults showed that skin and intestinal crypt could indeed be labeled (Fig. 2r-s’) consistent with the known *Lrig1* expression domain.

Then, we characterized the allele expression in the brain. *Lrig1* is expressed in many different cell types in the brain in addition to astrocytes [50–52]. The pattern of *Lrig1*^*T2A-iCreERT2*^ activity as visualized by the *ROSA26*^*Ai14*^ reporter matched the known expression pattern (Fig. 2t-t”). Of note, no neurons were labeled in the olfactory bulb at 3 days after tamoxifen injection (Fig. 2t”).

Next, we attempted to determine whether the sfGFP-iCre-ER^T2^ is co-expressed with Lrig1 in the V-SVZ cells. Interestingly, although we could readily detect sfGFP by indirect immunofluorescence with the anti-GFP antibody and confocal microscopy on brains from the *Cdk6*^*T2A-td-sfGFP*^ mouse line, the sfGFP-iCre-ER^T2^ from the *Lrig1*^*T2A-iCreERT2*^ allele was not detectable (Fig. 2u-u’). To establish that Lrig1 is expressed in the V-SVZ cells, we turned to a very sensitive *Lrig1*^*T2A-tdTomato*^ reporter allele utilizing the extremely bright red fluorescent protein tdTomato (similar to [53]). By flow cytometry, this mouse line revealed several discrete cell populations with tdTomato fluorescence from the *Lrig1* locus, demonstrating that Lrig1 is indeed expressed in the V-SVZ cells (Fig. 2u”). However, although we could again detect the sfGFP signal from the *Cdk6*^*T2A-td-sfGFP*^ allele by flow cytometry, we still could not detect any sfGFP signal from the *Lrig1*^*T2A-iCreERT2*^ allele (Fig. 2u”). These suggested that (1) Lrig1 protein expression level is low in the V-SVZ cells and (2) the sfGFP-iCre-ER^T2^ reporter fluorescence is not very sensitive either (as described above). Consistent with low Lrig1 protein level, an indirect immunofluorescence with an anti-Lrig1 antibody and confocal microcopy also did not reveal any signal in the V-SVZ (Fig. 2v).

Thus, we next utilized flow cytometry to detect and measure co-labeling of the RFP lineage label and the endogenous Lrig1. First, using the *Lrig1*^*T2A-tdTomato*^ allele we determined that the commercial anti-Lrig1 antibody indeed labels Lrig1-T2A-tdTomato+ cells (Fig. 2w). Conversely, all of the Lrig1-T2A-tdTomato+ cells in the *Lrig1*^*T2A-tdTomato/+*^ mice were labeled by the anti-Lrig1 antibody (Fig. 2w). Next, we detected the RFP+ cells in the tamoxifen-induced *Lrig1*^*T2A-iCreERT2/+*^*; ROSA26*^*Ai14/+*^ mice and observed that almost all of the RFP+ cells were labeled by the anti-Lrig1 antibody (Fig. 2x). This indicated that the RFP lineage labeling occurs only in Lrig1+ cells in the *Lrig1*^*T2A-iCreERT2/+*^*; ROSA26*^*Ai14/+*^ mice.

To characterize the kinetics of labeling, we injected tamoxifen into a cohort of 3 month-old *Lrig1*^*T2A-iCreERT2/+*^*; ROSA26*^*Ai14/+*^ mice once on the same day. Then, we analyzed at progressively longer time points the lateral walls from these mice by whole mount immunofluorescence and confocal imaging. When quantitated, the number of singlet cells plateaued at 7 days (Fig. 2y), indicating that the tamoxifen-induced in vivo labeling of cells with RFP was complete by day 7. That is, no additional cells were labeled after 7 days, but new RFP+ cells were born from the stem cells already labeled with RFP. Thus, although the cell number is under-sampled, day 3 is a good time point to assess the initially labeled cell population because the new cells are not born yet.

**Fig. 4:**
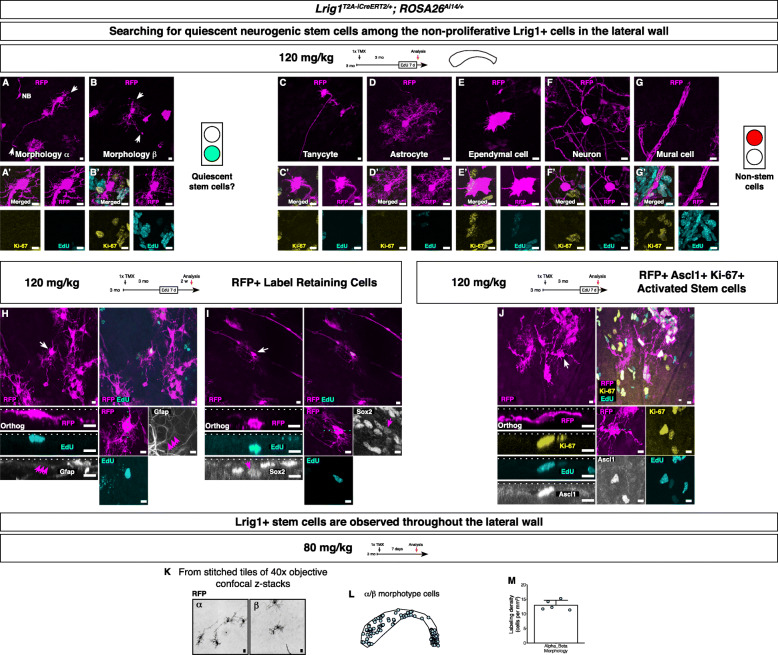
Identification of the Lrig1+ neurogenic stem cells. **a**-**b’** Potential stem cells labeled by the driver. **a**-**a’** α morphotype cell. Note the absence of Ki-67 and EdU signal after 1 week of EdU administration in drinking water. Note the branches and the long basal process (arrows). Note the RFP+ neuroblast (NB) nearby. Scale bar, 10 μm. **b**-**b’** β morphotype cell. Ki-67- and EdU-. Note the branches and short basal process. Scale bar, 10 μm. **c’**-**g’** Non-stem cells labeled by the driver. **c**-**c’** A tanycyte at the ventricular surface. Scale bar, 10 μm. **d**-**d’** A niche astrocyte deep in the lateral wall. Scale bar, 10 μm. **e**-**e’** An ependymal cell at the lateral surface. Scale bar, 10 μm. **f**-**f’** A striatal neuron deep in the lateral wall. Scale bar, 10 μm. **g**-**g’** A mural cell – a pericyte or a smooth muscle cell. Scale bar, 10 μm. **h**-**i** RFP+ EdU+ label retaining cells after 1 week of EdU administration and 2 weeks of chase. A Gfap+ RFP+ EdU+ cell. **h** and a Sox2+ RFP+ EdU+ cell. **i** Orthogonal views and zoomed views are below. Scale bar, 10 μm. **j** An RFP+ EdU+ Ascl1+ Ki-67+ singlet cell is shown (arrow). Note the large nucleus compared to the nearby cells. Orthogonal views and zoomed views are below. Scale bar, 10 μm. **k** Clear morphologies demonstrated in cells labeled sparsely and imaged at high resolution. Scale bar, 10 μm. **l** A representative whole mount showing the robust labeling of the α/β subtype cells throughout the lateral wall. **m** Labeling density of α/β subtype cells in the lateral wall. Mean ± standard deviation

We note that the same complement of cell types (see Fig. 4a-g’) was consistently labeled by the *Lrig1*^*T2A-iCreERT2*^ allele with the low (80 mg/kg) or high (230 mg/kg) tamoxifen doses. Using the *ROSA26*^*Ai14*^ reporter line [30], labeling of certain cell types could not be intentionally excluded by lowering the tamoxifen dose. Thus, despite the low Lrig1 level, all of the different Lrig1+ cell types in the V-SVZ expressed levels of the recombinase sufficient for *ROSA26*^*Ai14*^ reporter recombination at the tamoxifen doses utilized (80–230 mg/kg). There could nevertheless be a bias against labeling of cells with lower recombinase levels. For example, with very low tamoxifen doses (20–40 mg/kg), it was possible to obtain RFP labeling that was stochastically devoid of the rare Ki-67+ stem cells (data not shown), suggesting that the proliferating stem cells express lower level of Lrig1 and recombinase relative to the quiescent stem cells.

Finally, we compared our *Lrig1*^*T2A-iCreERT2*^ allele to the Coffey *Lrig1*^*creERT2*^ allele. The V-SVZ cell types labeled with our allele (see Fig. 4a-g’) were also labeled with the Coffey allele (data not shown). Taken together, these characterizations indicate that (1) the mice that carry the non-disruptive *Lrig1*^*T2A-iCreERT2*^ allele are indistinguishable from wildtype mice, (2) the *Lrig1*^*T2A-iCreERT2*^ allele faithfully reports Lrig1+ cells, and (3) the labeling by the *Lrig1*^*T2A-iCreERT2*^ allele is also indistinguishable from an independent mouse line from another laboratory utilizing the same *Lrig1* locus to drive the reporter gene.

### Consistent labeling of non-proliferative cells with the *Lrig1*^*T2A-iCreERT2*^ allele

To trace and quantitate the neurogenic output from the Lrig1+ cell lineages, we determined whether our knock-in allele allows consistent labeling. Three days after tamoxifen induction, whole mount immunofluorescence, confocal microscopy, and quantitation revealed consistent numbers of RFP+ cells labeled within each induction cohort and between different induction cohorts (Fig. 3a-b).

Next, because *Lrig1* is known to regulate quiescence in other stem cell systems, we determined whether non-proliferative cells are preferentially labeled with the *Lrig1* reporter mice. Ki-67, Mcm2, and Ascl1 are markers of proliferating cells in the V-SVZ. Whole mount immunofluorescence analysis on the lateral walls from the tamoxifen-induced *Lrig1* reporter mice revealed that among the RFP+ cells, Ki-67+ or Mcm2+ or Ascl1+ RFP+ cells were extremely rare (Fig. 3b), meaning most of the initially labeled RFP+ cells were non-proliferative.

### The neurogenic Lrig1+ cell lineage is active throughout adult life

Whether the non-proliferative Lrig1+ cells in the V-SVZ labeled by the *Lrig1*^*T2A-iCreERT2*^ allele possessed neurogenic activity throughout adult life was examined. Analysis of the olfactory bulbs 6 months (Fig. 3c-f) and 1 year (data not shown) after induction with a single 120 mg/kg tamoxifen dose revealed RFP+ granule cells (Fig. 3c) and RFP+ periglomerular cells (Fig. 3d). Counts of the RFP+ NeuN+ cells in the granular cell layer indicated more than a hundred RFP+ NeuN+ granule cells per 50 μm section (Fig. 3e-f). These suggested that proliferative activities arising from the non-proliferative RFP-labeled stem cells in the lateral wall gave rise to the RFP+ olfactory bulb interneurons.

Before further analysis, as a control, we determined whether adult neurogenesis was at a steady-state at the time of tamoxifen induction. We measured the Dcx+ pixel area in the lateral wall at progressively longer time points from the time of tamoxifen induction (*n* = 4–5 lateral walls from 4 to 5 mice per time point). These measurements revealed the Dcx+ pixel area did not increase or decrease from 3 months of age to 4 months of age (3 days to 1 month after injection, Fig. 3g), suggesting adult neurogenesis was at a steady-state at the time of tamoxifen induction. Generally consistent with a previous report [54], we observed a gradual decrease in the Dcx+ pixel area during aging, perhaps reflective of decrease in adult neurogenesis. This decrease in the Dcx+ pixel area first became statistically significant at 1 year of age.

To analyze the dynamics of newborn RFP+ Dcx+ cells in the lateral wall, the *Lrig1* reporter mice were induced once with a high dose of tamoxifen (230 mg/kg) at 3 months of age to label as many Lrig1+ cells as possible and perfused at progressively longer time points. The lateral walls were analyzed by whole mount immunofluorescence and confocal imaging (*n* = 4–5 lateral walls from 4 to 5 mice per time point). At day 3 after induction, very few RFP+ Dcx+ neuroblasts were detected (Fig. 3h). Significant numbers of RFP+ Dcx+ neuroblasts were first detected at 1 month post induction (mpi). At 3 mpi, the numbers of RFP+ Dcx+ neuroblasts had increased. By 6 mpi, the numbers of RFP+ Dcx+ neuroblasts had decreased. However, at 9 mpi, the numbers of RFP+ Dcx+ neuroblasts had stabilized. Notably, the numbers of RFP+ Dcx+ neuroblasts remained significant at even 1 year after induction, i.e., at 1 year and 3 months of age.

To analyze the dynamics of proliferating clusters, the *Lrig1* reporter mice were induced once at 3 months with a low dose of tamoxifen (80 mg/kg) to label the Lrig1+ stem cells at low density. The mice were perfused at progressively longer time points to analyze the lateral walls by whole mount immunofluorescence and confocal microscopy as above (*n* = 3–10 lateral walls from 3 to 5 mice per time point). The numbers of RFP+ cell clusters and the numbers of cells in each cluster of RFP+ cells (cluster size) were scored (Fig. 3i). First, the numbers of RFP+ cell clusters appeared cyclic: high at 1 mpi, low at 3 mpi, then high again at 9 and 12 mpi. Second, the mean cluster size remained similar between 1 mpi and 3 mpi although there were outliers of larger clusters at 1 mpi. From 3 mpi on, in atypical distributions, small (< 20 cells) clusters were present at all time points while the outlier cluster sizes increased from 6 mpi to 9 mpi, then decreased at 12 mpi.

Whether the neurogenic Lrig1+ cell lineage continues to contribute significantly to neurogenesis in the old mice was also determined. Analysis of the percentage of RFP+ Dcx+ cells pixel area over Dcx+ cells pixel area (Fig. 3j-j”) suggested that at 1 year after induction, 37.8 ± 10.8% of the Dcx+ cells arose from the Lrig1+ cell lineages (mean ± S.D., *n* = 4 lateral walls from 4 mice). In comparison, at 1 mpi, 32.3 ± 5.91% of the Dcx+ cells arose from the Lrig1+ cell lineages (mean ± S.D., *n* = 4 lateral walls from 4 mice).

### Heterogeneity among the Lrig1+ cells

The study of the Lrig1+ cell lineages in the lateral wall V-SVZ revealed robust neurogenic activity throughout adult life from this pool. As aforementioned, almost all of the RFP+ cells labeled by the *Lrig1*^*T2A-iCreERT2*^ allele were non-proliferative, suggesting that if any stem cells are labeled by this allele, they are likely to be in a quiescent state. Thus, we examined the non-proliferative RFP+ cells in greater detail using thymidine analog EdU to identify the quiescent neurogenic stem cells among the Lrig1+ cells. Lack of EdU incorporation (the EdU- cells) means that the cells had not gone through an S-phase and can be inferred to have remained quiescent, at least for the duration of the EdU administration. Because the RFP-labeled stem cells were activated in significant numbers starting at 1 mpi (Fig. 3h), the mice were induced once with tamoxifen at 3 months of age, then thymidine analog EdU was administered for 7 days at 3 months after induction to label all proliferating cells. The lateral wall whole mounts were analyzed for RFP and Ki-67 immunoreactivities and EdU incorporation. This revealed the entire repertoire of the RFP+ cells: EdU- Ki-67- quiescent cell types (Fig. 4a-g’) as well as EdU+ Ki-67+, Ascl1+, or EdU+ Ki-67+ Dcx+ proliferating cells (data not shown).

In sum, we identified two morphological subtypes of potential quiescent neurogenic stem cells among the RFP+ EdU- Ki-67- cells, categorized by morphometry of cell depth (superficial vs. deep), number of branches (more or less than 4), and the length of the basal process (short or long). We named these morphotypes of Lrig1+ cell lineages α (Fig. 4a-a’) and β (Fig. 4b-b’). The α and β morphotypes are similar in that they both have branches, but different in that the β morphotype has a shorter basal process. Other cell types that were also labeled (Fig. 4c-g’) were excluded as candidates for stem cells because (1) these cells are known not to be of neurogenic stem cell lineages, and (2) we did not observe proliferating clusters of these cells during our experiments We counted from 4 lateral walls from 4 mice: 297 α morphotype cells; 159 β morphotype cells; 15 tanycytes; 12 striatal astrocytes; 219 ependymal cells; 3 neurons; 162 mural cells.

### Cell cycle entry of the Lrig1+ neurogenic stem cells

Having identified the potential quiescent Lrig1+ stem cells in the V-SVZ, we again used proliferation markers, this time to identify the Lrig1+ cells that proliferate and give rise to neurogenic progeny – the minimum criteria for a neurogenic stem cell identity. The tamoxifen induced *Lrig1* reporter mice were administered EdU for 7 days at 3 mpi. Then, the mice were perfused after a 14 days chase. After the chase, the EdU-labeled neuroblasts had migrated out of the V-SVZ to the olfactory bulbs, and sparse EdU-labeled stem cells (cells that had previously entered S-phase) remained in the lateral wall (Fig. 4h-i, 5161 cells counted, 5 lateral walls from 4 mice). Among these EdU+ cells, we observed very rare EdU+ RFP+ cells with the α/β morphologies (Fig. 4h-i, 21 cells counted, 5 lateral walls from 4 mice), suggesting that the RFP+ cells with these morphologies are the only Lrig1+ cells that can enter the cell cycle. We did not observe any label retention among any other RFP+ non-stem cell types (i.e., the cells in Fig. 4c-g’, 5 lateral walls from 4 mice), suggesting that these cells do not proliferate often at steady-state at this age. We also infer that these cells cannot be the neurogenic stem cells.

The retention of EdU label only in the α/β morphotype cells meant that only these cells had entered S-phase during the EdU administration, fulfilling a criterion for a stem cell identity. Thus, we searched for the Lrig1+ stem cells at an earlier phase of neurogenesis. Specifically, we looked for EdU+ Ki-67+ Ascl1+ RFP+ activated stem cells before the 2 weeks of chase. The tamoxifen induced *Lrig1* reporter mice were administered EdU for 7 days, then analyzed immediately after. Again, the non-stem cell types were not labeled with EdU, and we reiterate that most EdU-labeled cells were EdU+ Ascl1+ RFP+ transit amplifying cells and EdU+ Dcx+ RFP+ neuroblasts. However, we observed very rare singlet RFP+ cells with α/β morphologies that were EdU+ Ascl1+ and Ki-67+ (Fig. 4j, 38 cells counted from 7 lateral walls from 7 mice), suggesting that these cells are the quiescent RFP+ cells that entered the cell cycle to proliferate. Expression of Ascl1 demonstrated that these activated stem cells were in the neurogenic lineage. The singlet EdU+ Ascl1+ Ki-67+ RFP+ cells showed large nuclei, suggesting that the increased nuclear size is a characteristic of the activated neurogenic stem cells. The percentage of the RFP-labeled activated stem cells among all RFP-labeled stem cells was ~ 2.3% (~ 4.2 EdU+ RFP+ label-retaining cells per lateral wall, ~ 5.4 EdU+ Ascl1+ Ki-67+ RFP+ cells with large nucleus per lateral wall, ~ 211 RFP+ α/β morphotype cells per lateral wall).

### Spatial distribution of the Lrig1+ neurogenic stem cells in the lateral wall

The analysis above suggested that the RFP+ α/β morphotype cells could be the Lrig1+ quiescent neurogenic stem cells. To determine whether the Lrig1 expression identifies all spatial subtypes of the V-SVZ neurogenic stem cells [55], we analyzed the spatial distribution of the α/β morphotype stem cells in the lateral wall. Mice were induced sparsely such that the full morphology of each cell could be visualized without compromise. After whole mount immunofluorescence, we performed high magnification imaging of the entire lateral walls (Fig. 4k, Additional file 3). The α/β morphotype stem cells (Additional file 4) were observed throughout the lateral wall (Fig. 4l-m, *n* = 5 mice), suggesting that all spatial subtypes of neurogenic stem cells are labeled by this driver.

### Ara-C infusion induced activation of the Lrig1+ neurogenic stem cells

The RFP+ cells of the α/β morphotypes were the only cells observed to enter the cell cycle, suggesting that these cells are the Lrig1+ quiescent neurogenic stem cells. Furthermore, these cells were observed throughout the lateral wall. We further tested the quiescence and subsequent activation of the α/β morphotype stem cells via infusion of the nucleoside anti-mitotic Ara-C. As a classical chemotherapeutic, Ara-C infusion kills the dividing neurogenic cells in the V-SVZ, and concomitantly induces regeneration of the neurogenic lineage from the remaining qNSC’s [56]. In our implementation of this paradigm, we infused Ara-C into the cerebrospinal fluid of the lateral ventricle [15] rather than into the cortical parenchyma [56] for more rapid kinetics. In our implementation, temporary up-regulation of Gfap in the ependymal cells was observed, but olfactory bulb interneurons were nevertheless generated from the V-SVZ after the infusion, as determined by thymidine analog pulse-chase.

**Fig. 5:**
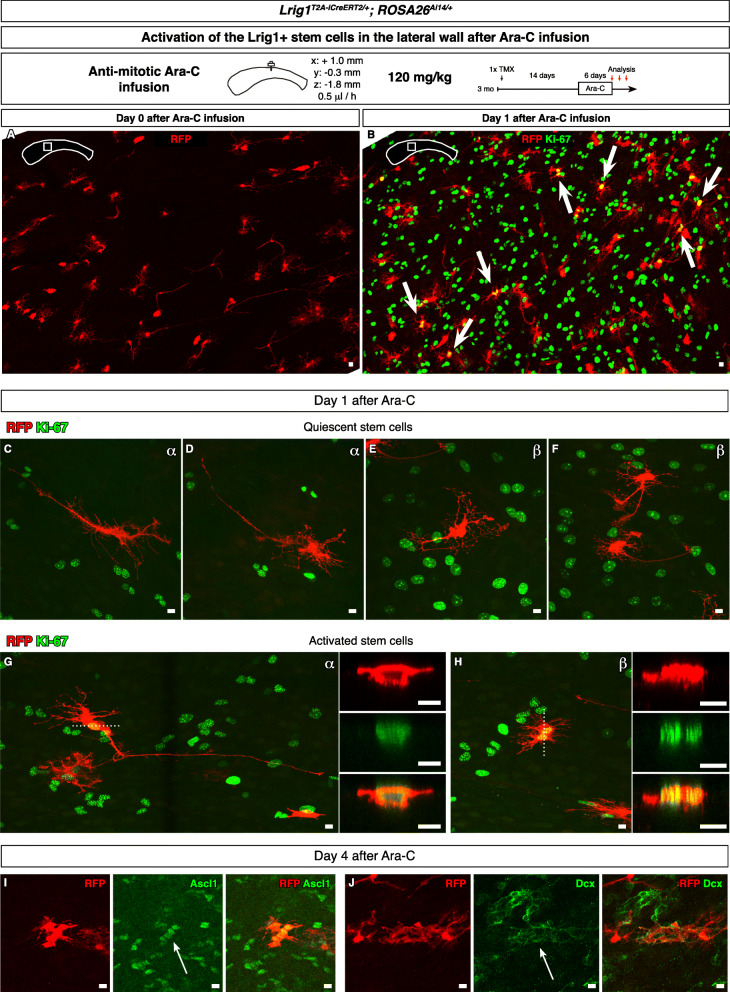
Activation of the Lrig1+ neurogenic stem cells after Ara-C infusion. **a** A whole mount from a mouse infused Ara-C for 6 days. Scale bar, 10 μm. **b** A whole mount from a mouse infused Ara-C then chased for 1 day. Scale bar, 10 μm. **c**-**f** Even after the Ara-C infusion, some RFP+ cells did not enter the cell cycle. **c**-**d** α morphotype cells that had remained quiescent. Scale bar, 10 μm. **e**-**f** β morphotype cells that had remained quiescent. Scale bar, 10 μm. **g** However, some cells did enter the cell cycle. An α morphotype cell that entered the cell cycle as evident by positive Ki-67 immunoreactivity. Scale bar, 10 μm. **h** β morphotype cells that had entered the cell cycle, and apparently divided into two cells, also positive for Ki-67. Scale bar, 10 μm. **i**-**j** RFP+ cells clusters at 4 days after Ara-C infusion. The cells in clusters were immunoreactive for Ascl1 (**i**) or Dcx (**j**). Scale bar, 10 μm

We labeled the Lrig1+ cells with a single injection of tamoxifen. Fourteen days after the induction, mice were infused with Ara-C for 6 days. After the infusion, whole mount immunofluorescence and confocal imaging of the lateral wall revealed RFP+ cells that survived the infusion and persisted (Fig. 5a, 1458 α/β morphotype cells counted from 6 lateral walls from 6 mice), indicating that these RFP+ cells were quiescent. Among these quiescent cells would be the quiescent stem cells.

To identify the quiescent neurogenic stem cells, in another cohort of mice, we ceased the infusion after 6 days then analyzed the lateral wall after waiting a day when the RFP+ quiescent stem cells were activated to participate in the subsequent regeneration (Fig. 5b). Interestingly, we observed evenly spaced Ki-67+ nuclei at this time point. Among the RFP+ cells, some but not all were also Ki-67+ (Fig. 5b-f). Singlet Ki-67+ RFP+ cells showed large nuclei. The numbers of Ki-67+ RFP+ cells were increased over the uninfused mice at steady-state (Ara-C-induced, 219 cells counted from 3 lateral walls from 3 mice; steady-state, 38 cells counted from 7 lateral walls from 7 mice). The Ki-67+ RFP+ cells were exclusively of the α/β morphotype (Fig. 5g-h), indicating that only these subtypes could enter the cell cycle in response to the Ara-C infusion. The Ki-67+ RFP+ cells were observed throughout the anterior-posterior and dorsal-ventral axes (data not shown). We did not observe any other RFP+ cell types with Ki-67 expression.

At later time points, clusters of RFP+ cells that co-labeled with the neurogenic lineage markers Ascl1 (Fig. 5i) and Dcx (Fig. 5j) could be observed, indicating that the Ara-C infusion-associated activation of the RFP+ quiescent stem cells results in neurogenic progeny and not simply glial scarring. Thus, the Lrig1+ α/β morphotype cells could enter the cell cycle from quiescence and generate neurogenic progeny, i.e., they fulfilled the minimum criteria for a neurogenic stem cell identity.

### Location of the Lrig1+ neurogenic stem cells throughout the depth of the lateral wall

**Fig. 6:**
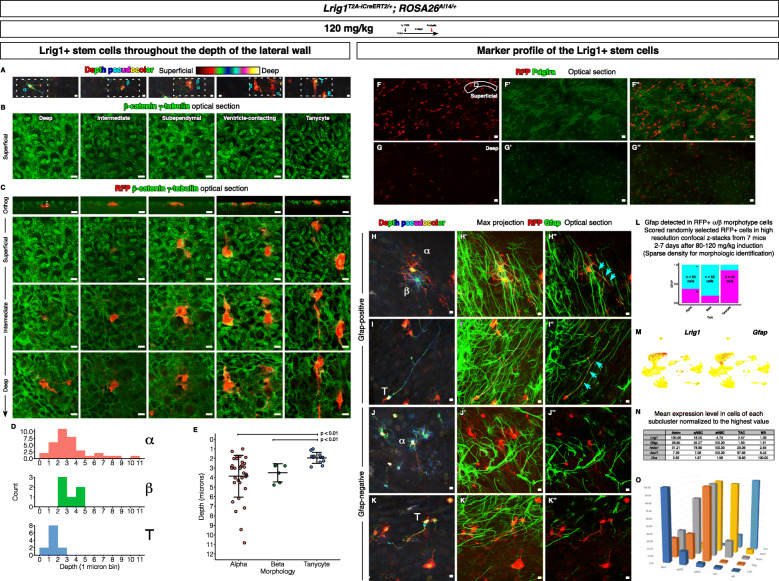
Characteristics of the Lrig1+ neurogenic stem cells. **a** The cells are shown in depth pseudocolor maximum projection to reveal their location in the lateral wall. E, ependymal cell. Scale bar, 10 μm. **b** Ventricular surface as revealed by the β-catenin and γ-tubulin staining. Scale bar, 10 μm. **c** The different types of RFP+ cells are shown at different depths of the lateral wall. Scale bar, 10 μm. **d** Histogram of distance between cell center and ventricular surface measured from orthogonal views of high resolution confocal z-stacks (see orthogonal view in **c**). **e** Dot plot of the distance between cell center and ventricular surface. Mean ± standard deviation. **f**-**g”** Lrig1+ cell lineages and Pdgfra. **f**-**f”** RFP and Pdgfra at the ventricular surface. Scale bar, 10 μm. **g**-**g”** RFP and Pdgfra deep in the lateral wall. Scale bar, 10 μm. **h**-**k”** Lrig1+ cell lineages and Gfap. **h**, **i**, **j**, **k** The cells are pseudo-colored based on the distance from the ventricular surface to highlight their position in the lateral wall. **h**-**h”** Gfap+ α/β subtype cells. Scale bar, 10 μm. **i**-**i”** Gfap+ tanycytes. Scale bar, 10 μm. **j**-**j”** Gfap- α/β subtype cells. Scale bar, 10 μm. **k**-**k”** Gfap- tanycytes. Scale bar, 10 μm. **l** Percentage of Gfap protein in α/β subtype cells and tanycytes. **m** *Lrig1* and *Gfap* in single cell RNA sequencing clusters. **n** Mean of expression levels in cells of each subcluster. **o** Graph of the mean expression levels

We determined whether the Lrig1+ neurogenic stem cells show phenotypes consistent with known V-SVZ stem cells. *Lrig1* reporter mice were induced as above. Whole mount immunofluorescence, confocal imaging analyses (Fig. 6a-c), and measurements of z-stacks (in orthogonal view, the distance between the center of the RFP+ cell and the ventricular surface revealed by β-catenin immunoreactivity, Fig. 6c) revealed that, on average, the α/β morphotype stem cells were located deep in the lateral wall, but they were found throughout the depth of the lateral wall (Fig. 6d-e). As a comparison, the ventricle-contacting tanycytes were analyzed. As expected, these cells always contacted the ventricular surface. The differences in the cells’ locations between the α/β morphotype cells and tanycytes were statistically significant (Fig. 6e, α vs. tanycytes, *p* < 0.01, β vs. tanycytes, *p* < 0.05, t test).

### Marker profile of the Lrig1+ neurogenic stem cells

Finally, the expression of known markers in the Lrig1+ neurogenic stem cells were characterized. Since the Lrig1+ α/β morphotype stem cells resemble oligodendrocyte precursor cells (OPC’s) morphologically, we examined a canonical marker of the OPC’s, Pdgfra [57], in lateral walls from tamoxifen-induced *Lrig1* reporter mice. In the V-SVZ, the Pdgfra immunostaining revealed (1) signal near the ventricular surface (Fig. 6f') and (2) signal deep in the lateral wall (Fig. 6g'). At the surface, the RFP+ Dcx+ neuroblasts co-labeled with Pdgfra (Fig. 6f”). However, deeper in the wall, RFP did not co-label at all with Pdgfra (i.e., the OPC’s, Fig. 6g”), indicating that the Lrig1+ α/β morphotype stem cells are not OPC’s.

Next, analyses of the Gfap protein in the lateral walls from the tamoxifen-induced *Lrig1* reporter mice (Fig. 6h-k”) surprisingly revealed only infrequent expression in the Lrig1+ stem cells (Fig. 6l). More than half of the α/β morphotype stem cells labeled by the *Lrig1* reporter did not express the Gfap protein. In contrast, nearly all cells in the control group of tanycytes expressed the Gfap protein (Fig. 6l). Interestingly, this heterogeneity of Gfap expression was also suggested in the single cell RNA sequencing analysis. *Gfap* expression was scattered and non-uniform in the astrocyte and the qNSC clusters (Fig. 6m). Furthermore, the calculation of the mean *Gfap* expression level showed that the *Gfap* expression level was highest in the activated stem cells (Fig. 6n-o).

## Discussion

Here, we have identified *Lrig1* as a potential marker of stem cells in the V-SVZ neurogenic lineage and analyzed the Lrig1+ neurogenic stem cells in vivo.

Our logic for assigning a neurogenic stem cell identity to the α/β morphotype cells defined by the *Lrig1*^*T2A-iCreERT2*^ allele is the following. First, we observed robust numbers of RFP+ Dcx+ neuroblasts and neurons at later time points. Second, the labeling of more differentiated RFP+ and Ki-67+, Mcm2+, Ascl1+, or Dcx+ cells were negligible at early time points, indicating a very narrow labeling early in the neurogenic lineage. Third, cell cycle entry was detected only in the α/β morphotype cells among all of the non-proliferative cell types labeled in the *Lrig1* reporter mouse. Thus, the pool of RFP+ cells gave birth to neuroblasts and neurons, and the only RFP+ cells that we could detect going into the cell cycle were the α/β morphotype cells. Because no other RFP+ cell types were proliferating, we conclude that the α/β morphotype cells must be the neurogenic stem cells. In support of this conclusion, inducing activation by Ara-C infusion resulted in cell cycle entry of only the α/β morphotype cells. Furthermore, consistent with the increased need for regeneration from stem cells after Ara-C infusion, the number of RFP+ α/β morphotype cells that were Ki-67+ was increased over the number of these cells that were Ki-67+ at steady-state. Finally, consistent with the notion that Lrig1 expression identifies all spatial subtypes of neurogenic stem cells, the α/β morphotype cells were observed throughout the lateral wall.

Interestingly, the Lrig1+ neurogenic stem cells appear similar to the branched qNSC first described by Codega et al. [12], suggesting that they could be the same type of stem cells. Morphologically, these cells also show branches and a basal process, and often but not always contact the ventricle. Molecularly, the qNSC’s in Codega et al. were also enriched for *Lrig1* and other genes also expressed in the Lrig1+ neurogenic stem cells. However, many of the Lrig1+ neurogenic stem cells did not express Gfap. The reason for this discrepancy remains mysterious, but could include the detection limit of our imaging, the activity of the *hGFAP* promoter in mice, as well as peculiarities of the Gfap protein expression.

In addition to serving as a marker, *Lrig1* is functionally relevant to V-SVZ neurogenesis because *Lrig1* knock-out mice show increased proliferation in the V-SVZ in line with previous studies [19, 49]. We are currently studying this phenotype in greater detail. Because Lrig1 is a membrane protein with an extracellular domain, this implies that inhibition of the Lrig1 protein activity with an antibody (analogous to immune checkpoint inhibitor blockade) might be an approach to increase neurogenic activity from these stem cells.

Finally, the existence of long-lived Lrig1+ neurogenic stem cells raises the question of how these cells are allocated genetically and epigenetically. Fortuitously, Lrig1 itself regulates stem cell proliferation. Thus, the analysis of *Lrig1* mutants in the future may reveal whether the adult neurogenic stem cell pool can be modulated by modifying *Lrig1* expression during development.

## Conclusions

We conclude that the EdU- Ki-67- Mcm2- Ascl1- α/β morphotype cells in the lateral wall defined by the *Lrig1*^*T2A-iCreERT2*^ allele are stem cells that are neurogenic throughout adult life. The gene we identified as a marker of these neurogenic stem cells may be an important regulator of adult neural stem cell proliferation as well as a genetic determinant of the adult neural stem cell pool size.

## Supplementary information

**Additional file 1 **Identification of a candidate gene *Lrig1* from the Id1^high^ neural stem cells. **a** Id1-Venus knock-in allele design from [15]. **b** The Id1-Venus fluorescence responds to changes in exogenous factors such as FGF-2, EGF, and BMP4. **c** Expression of neurogenic marker gene Ascl1 and oligodendrogliogenic marker gene Olig2 in different cell fractions from these cells. **d** Diagram of the cells FACS sorted for transcript analysis. **e-e”** *Lrig1* was more highly expressed in the Id1^high^ cell fraction. Mean ± standard deviation.

**Additional file 2 **Antibody validations in whole mount immunofluorescence. **a-a”’** Anti-Ascl1 and anti-Dcx antibodies were validated against GFP from *Cdk6*^*T2A-td-sfGFP*^ allele. All GFP+ cells were either Ascl1+ or Dcx+. Scale bar, 100 μm. **b-b”’** Anti-Dcx and anti-Ki-67 antibodies were validated against proliferating cells’ nuclei labeled by 7 days administration of thymidine analog EdU. All Ki-67+ cells were labeled by the immunofluorescence procedure. In addition, the EdU dose only minimally affected neurogenic cells, as evidenced by the low number of pyknotic cells in the lateral wall (arrows). Scale bar, 100 μm. **c-c”’** Antibody against Mcm2 [58] was validated against anti-Ki-67 antibody staining (that was validated above). The immunostaining with the two antibodies were virtually identical. Note that Ki-67+ Mcm2+ RFP+ cells were only rarely observed at this early time point after tamoxifen induction. Scale bar, 100 μm.

**Additional file 3.** All RFP+ cells labeled by the reporter allele at an early time point. A lateral wall processed 7 days after low dose tamoxifen induction. Note the clear demonstration of distinct cell types described in Fig. 4. Scale bar, 100 μm.

**Additional file 4.** Additional examples of Lrig1+ neurogenic stem cells with the α/β morphologies. A lateral wall processed 3 days after tamoxifen induction. Note the variations on a theme of cell body with branches and a basal process. Scale bar, 10 μm.

**Additional file 5.** The R script utilized to analyze the single cell RNA sequencing data.

## Data Availability

The datasets generated and/or analyzed during the current study are not publicly available due to file sizes but are available from the corresponding author on reasonable request.

## Abbreviations

Agt: Angiotensinogen
aNSC: Activated neural stem cell
Ascl1: Achaete-scute family bHLH transcription factor 1
BSA: Bovine serum albumin
Cdk6: Cyclin-dependent kinase 6
Dcx: Doublecortin
EdU: Ethynyl deoxyuridine
EGF: Epidermal growth factor
EGFR: Epidermal growth factor receptor
Gfap: Glial fibrillary acidic protein
Lrig1: Leucine-rich repeats and immunoglobulin-like domains 1
Mcm2: Minichromosome maintenance complex component 2
NB: Neuroblast
NeuN: Neuronal nuclei
Nr2e1: Nuclear receptor subfamily 2, group E, member 1
OPC: Oligodendrocyte precursor cell
PBS: Phosphate-buffered saline
Pdgfra: Platelet-derived growth factor receptor alpha
PLP fixative: Paraformaldehyde-Lysine-Periodate fixative
qNSC: Quiescent neural stem cell
SGZ: Subgranular zone
TAC: Transit amplifying cell
V-SVZ: Ventricular-subventricular zone
