# Correction: Lrig1 expression prospectively identifies stem cells in the ventricular-subventricular zone that are neurogenic throughout adult life

**DOI:** 10.1186/s13064-023-00171-1

**Published:** 2023-05-19

**Authors:** Hyung-song Nam, Mario R. Capecchi

**Affiliations:** Department of Human Genetics, University of Utah School of Medicine, Salt Lake City, UT 84112‑5331 USA

**Correction: Neural Dev 15, 3 (2020)**

10.1186/s13064-020-00139-5

The authors would like to correct errors in the original publication of the article [1].

1. Page 2, “the G4 embryonic stem cells derived from 129S6/SvEvTac × C57BL/6Ncr F1 embryos” corrected to “the G4 embryonic stem cells derived from a 129S6/SvEvTac × C57BL/6Ncr F1 embryo”.

2. Page 6, “In addition to our line of investigation (Additional file 1), others have also previously observed *Lrig1* expression in the V-SVZ quiescent neural stem cells (qNSC’s) [12]” corrected to “In addition to our line of investigation (Additional file 1), others have also previously observed *Lrig1* expression in the V-SVZ quiescent neural stem cells (qNSC’s) [2, 3]”. An additional reference was added. See references below.

3. Page 7, “When heterozygotes of this C57BL/6J congenic mouse line were interbred” corrected to “When heterozygotes of this C57BL/6J congenic mouse line were intercrossed”.

4. Page 9, “consistent with the known *Lrig1* expression domain” corrected to “consistent with the known *Lrig1* expression domains”.

5. Page 11, Fig. 4 legend, “An ependymal cell at the lateral surface” corrected to “An ependymal cell at the ventricular surface”.

6. Page 13, “as well as EdU + Ki-67 + , Ascl1 + , or EdU + Ki-67 + Dcx + proliferating cells (data not shown)” corrected to “as well as EdU + Ki-67 + Ascl1 + or EdU + Ki-67 + Dcx + proliferating cells (data not shown)”.

7. Page 13, “very rare singlet RFP + cells with α/β morphologies that were EdU + Ascl1 + and Ki-67 + (Fig. 4j, 38 cells counted from 7 lateral walls from 7 mice)” corrected to “very rare singlet RFP + cells with α/β morphologies that were EdU + Ascl1 + Ki-67 + (Fig. 4j, 38 cells counted from 7 lateral walls from 7 mice)”.

8. Page 13, “(2) we did not observe proliferating clusters of these cells during our experiments” corrected to “(2) we did not observe proliferating clusters of these cells during our experiments.”. A missing period was added.

9. Page 14, “(Fig. 6e, α vs. tanycytes, p < 0.01, β vs. tanycytes, p < 0.05, t test)” was corrected to “(Fig. 6e, α vs. tanycytes, p < 0.01, β vs. tanycytes, p < 0.01, t test)”. One of the two p-values was corrected.

10. Page 16, Fig. 6 legend, “Gfap- α/β subtype cells” corrected to “Gfap- α subtype cell”.

11. Page 16, Fig. 6 legend, “Gfap + tanycytes” corrected to “Gfap + tanycyte”.

12. Page 16, Fig. 6 legend, “Gfap- tanycytes” corrected to “Gfap- tanycyte”.

13. Page 17, Additional file legend, “such as FGF-2, EGF, and BMP4” corrected to “such as FGF2, EGF, and BMP4”.
