# Supplementary material for: Lrig1 expression prospectively identifies stem cells in the ventricular-subventricular zone that are neurogenic throughout adult life
Source: Neural Dev. 2020 Mar 17;15:3. doi: 10.1186/s13064-020-00139-5 (PMC7077007; doi:10.1186/s13064-020-00139-5)

Additional file 1

Lrig1 in Id1^{high} neural stem cells cultured in vitro

Adapted from Nam and Benezra, 2009

A

Id1-VenusYFP fusion protein

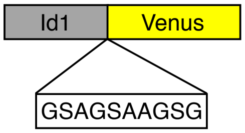

Id1^{Venus} knock-in allele

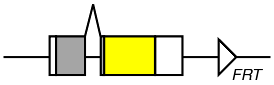

Establish NSPC cultures with unfractionated lateral wall cells of *Id1$^{Venus/Venus}$* mice using FGF-2 and EGF

B The Id1-Venus signal in these cells responds to exogenous factors (~24 h after changing)

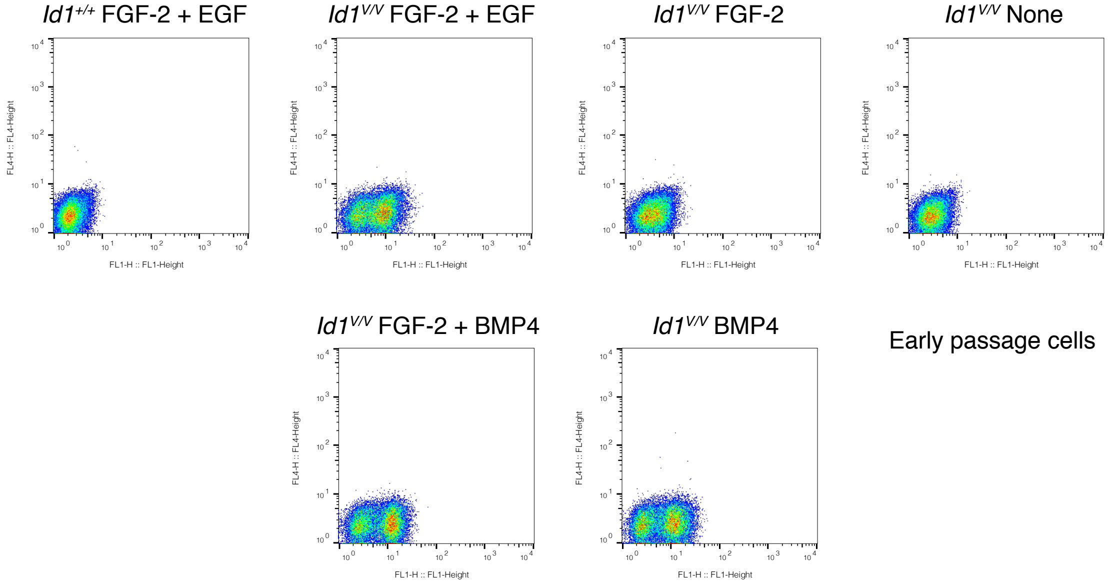

C Marker protein expression in cultured *Id1$^{V/V}$* NSPC fractions (+FE)

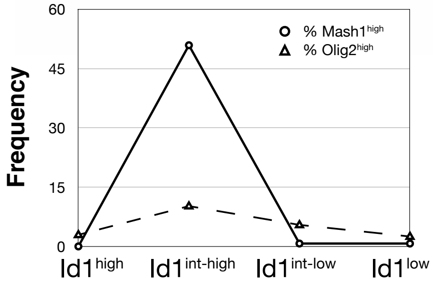

D FACS sort of Id1^{high} fraction (+FE)

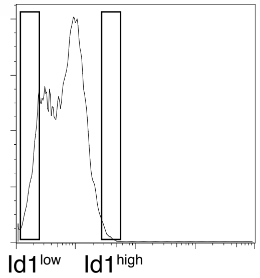

→ Microarray analyses of RNA from Id1^{high} and Id1^{low} fractions of *Id1$^{V/V}$* NSPC cultured with FGF-2 + EGF
→ Transcripts >1.5-fold enriched in the Id1^{high} fraction
→ Candidate genes for further studies, *Lrig1*, *Cdk6*, *Clu*, etc

E

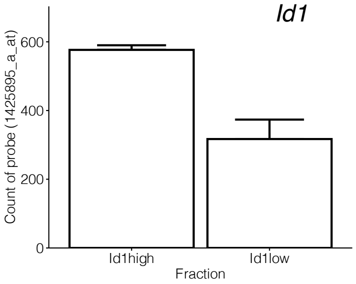

E'

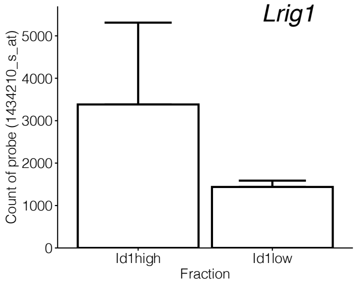

E''

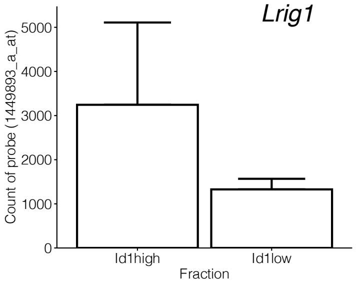

Supplement: Supplementary file 1 — Additional file 1 Identification of a candidate gene Lrig1 from the Id1high neural stem cells. a Id1-Venus knock-in allele design from [15]. b The Id1-Venus fluorescence responds to changes in exogenous factors such as FGF-2, EGF, and BMP4. c Expression of neurogenic marker gene Ascl1 and oligodendrogliogenic marker gene Olig2 in different cell fractions from these cells. d Diagram of the cells FACS sorted for transcript analysis. e-e” Lrig1 was more highly expressed in the Id1high cell fraction. Mean ± standard deviation. [file 13064_2020_139_MOESM1_ESM.pdf]
