# Supplementary figures and images for: Lrig1 expression prospectively identifies stem cells in the ventricular-subventricular zone that are neurogenic throughout adult life
Source: Neural Dev. 2020 Mar 17;15:3. doi: 10.1186/s13064-020-00139-5 (PMC7077007; doi:10.1186/s13064-020-00139-5)

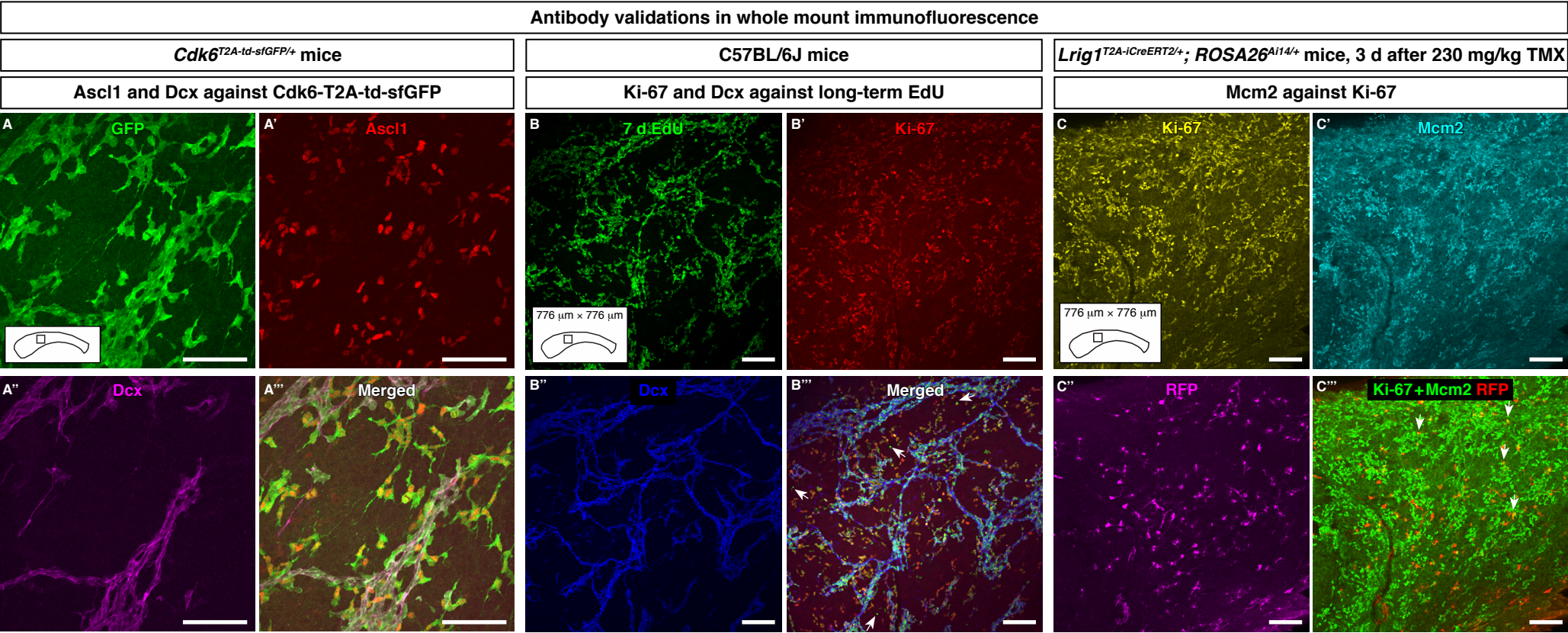

Supplement: Supplementary file 2 — Additional file 2 Antibody validations in whole mount immunofluorescence. a-a”’ Anti-Ascl1 and anti-Dcx antibodies were validated against GFP from Cdk6T2A-td-sfGFP allele. All GFP+ cells were either Ascl1+ or Dcx+. Scale bar, 100 μm. b-b”’ Anti-Dcx and anti-Ki-67 antibodies were validated against proliferating cells’ nuclei labeled by 7 days administration of thymidine analog EdU. All Ki-67+ cells were labeled by the immunofluorescence procedure. In addition, the EdU dose only minimally affected neurogenic cells, as evidenced by the low number of pyknotic cells in the lateral wall (arrows). Scale bar, 100 μm. c-c”’ Antibody against Mcm2 [58] was validated against anti-Ki-67 antibody staining (that was validated above). The immunostaining with the two antibodies were virtually identical. Note that Ki-67+ Mcm2+ RFP+ cells were only rarely observed at this early time point after tamoxifen induction. Scale bar, 100 μm. [file 13064_2020_139_MOESM2_ESM.pdf]
