# Supplementary material for: Lrig1 expression prospectively identifies stem cells in the ventricular-subventricular zone that are neurogenic throughout adult life
Source: Neural Dev. 2020 Mar 17;15:3. doi: 10.1186/s13064-020-00139-5 (PMC7077007; doi:10.1186/s13064-020-00139-5)

Additional file 3

***Lrig1**$^{T2A\text{-}iCreERT2/+}$**; ROSA26**$^{Ai14/+}$*

RFP+ cells labeled by the *Lrig1*$^{T2A\text{-}iCreERT2}$ allele at an early time point after low dose tamoxifen induction

80 mg/kg

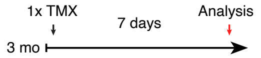

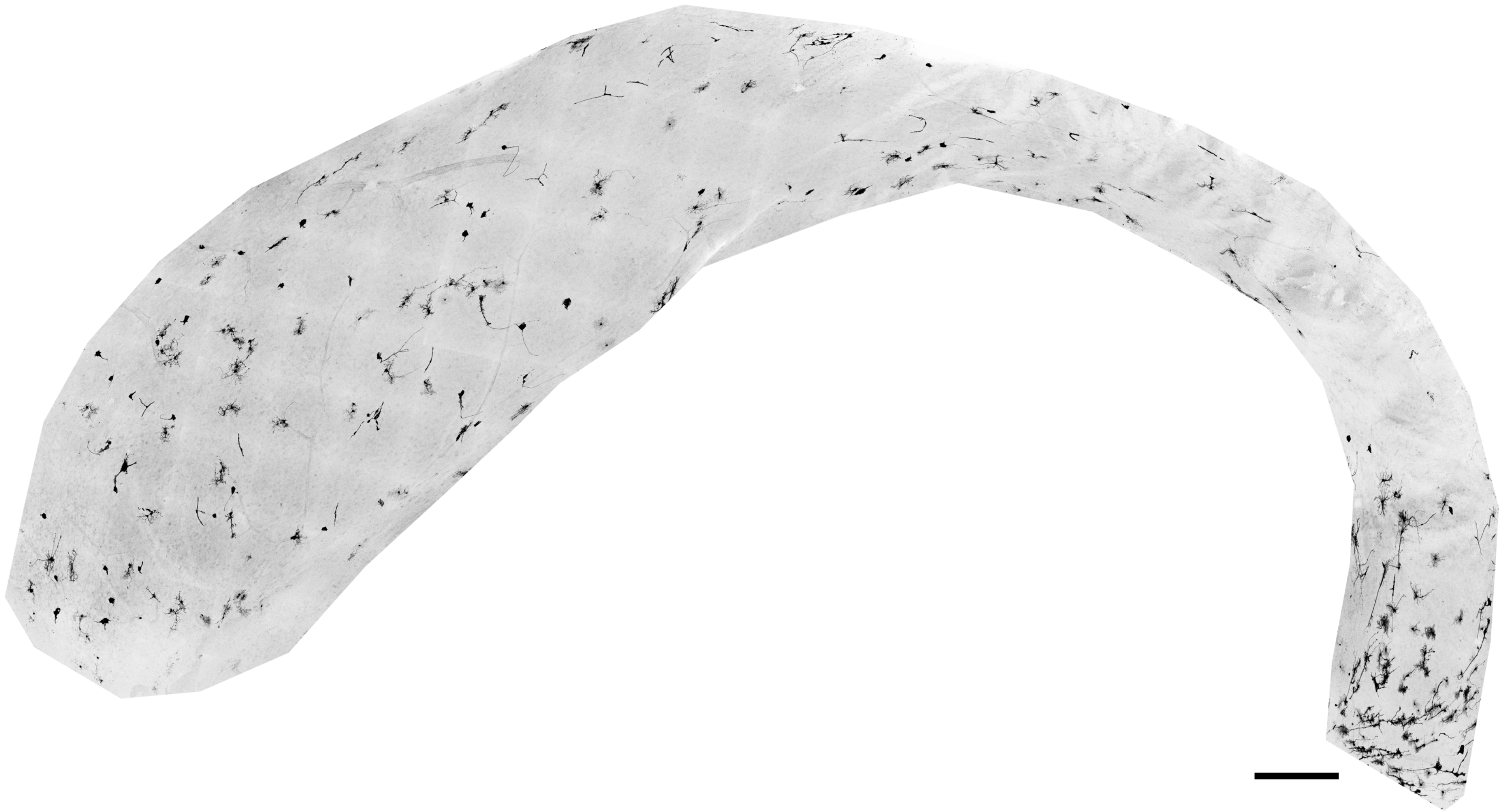

Supplement: Supplementary file 3 — Additional file 3. All RFP+ cells labeled by the reporter allele at an early time point. A lateral wall processed 7 days after low dose tamoxifen induction. Note the clear demonstration of distinct cell types described in Fig. 4. Scale bar, 100 μm. [file 13064_2020_139_MOESM3_ESM.pdf]
