# Supplementary material for: Lrig1 expression prospectively identifies stem cells in the ventricular-subventricular zone that are neurogenic throughout adult life
Source: Neural Dev. 2020 Mar 17;15:3. doi: 10.1186/s13064-020-00139-5 (PMC7077007; doi:10.1186/s13064-020-00139-5)

Additional file 4

$Lrig1^{T2A\text{-}iCreERT2/+}$; $ROSA26^{Ai14/+}$

Lrig1+ neurogenic stem cells with α/β morphologies

120 mg/kg

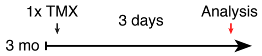

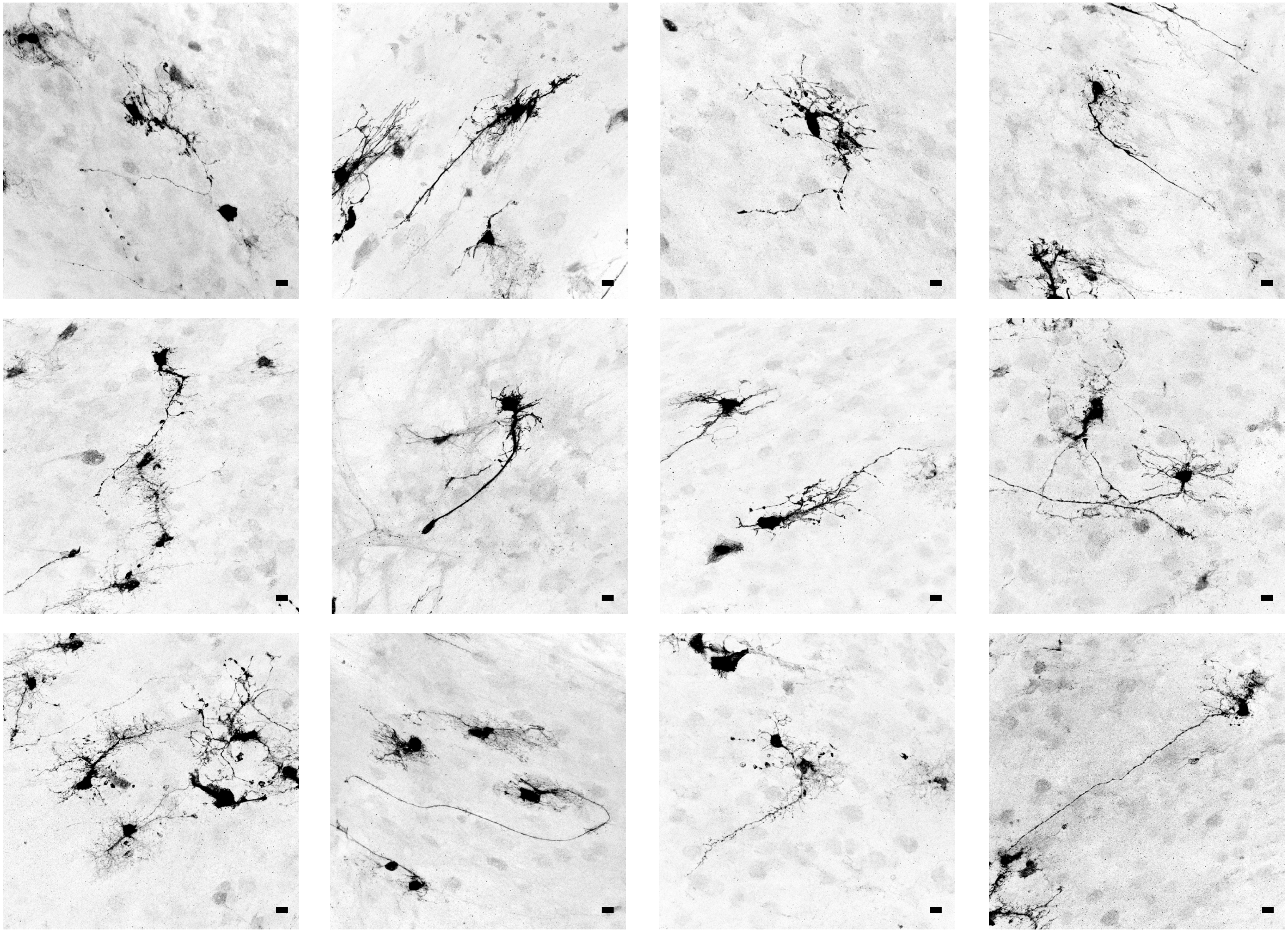

Supplement: Supplementary file 4 — Additional file 4. Additional examples of Lrig1+ neurogenic stem cells with the α/β morphologies. A lateral wall processed 3 days after tamoxifen induction. Note the variations on a theme of cell body with branches and a basal process. Scale bar, 10 μm. [file 13064_2020_139_MOESM4_ESM.pdf]
