# Supplementary material for: Lrig1 expression prospectively identifies stem cells in the ventricular-subventricular zone that are neurogenic throughout adult life
Source: Neural Dev. 2020 Mar 17;15:3. doi: 10.1186/s13064-020-00139-5 (PMC7077007; doi:10.1186/s13064-020-00139-5)
Supplement: Supplementary file 5 — Additional file 5. The R script utilized to analyze the single cell RNA sequencing data. [file 13064_2020_139_MOESM5_ESM.pdf]

Additional file 5

```
# A script to analyze the dataset in Mizrak et al., 2019
# Hyung-song Nam, PhD 2019

#####################

library(reticulate)
library(Seurat)
library(ggplot2)
library(dplyr)
library(gplots)
library(monocle)

############

theme_set(theme_cowplot())

########################

# download the counts files from the NCBI database
# the file names need to be reformatted to fit with this script
# see below

to_analyze <- c("GSE109447_13055_cells", "GSE109447_29319_cells")

for (data_sets in to_analyze ){

  counts <- paste(data_sets,"_matrix.txt", sep="")
  names <- paste(data_sets,"_id.txt", sep="")

  counts_file_2 <- read.table(file=counts, header = FALSE, sep="\t")

  name_file_2 <- read.table(file=names, header = FALSE)

  counts_file_2 <- counts_file_2[!duplicated(counts_file_2[,2]),]

  row.names(counts_file_2) <- as.character(counts_file_2[,2])

  colnames(counts_file_2) <- as.character(name_file_2$V1)

  counts_file_2 <- counts_file_2[,-c(1,2)]

  ###########################

  plate_barcodes <- c("latRep")

  rx_plate_barcodes <- glob2rx(paste("*",plate_barcodes,"*",sep=""))

  list_of_cell_names <- colnames(counts_file_2)

  list_of_grepped_cell_names <-
    subset(list_of_cell_names, grepl(paste(rx_plate_barcodes, collapse="|"),
                                     list_of_cell_names) )

  counts_file_2 <- counts_file_2[, list_of_grepped_cell_names]

  ##########
```

```
  ERCC <- glob2rx(paste("*","CRERECOMB","*",sep=""))

  gene_names <- row.names(counts_file_2)

  ERCC_genes <-
    subset(gene_names, grepl(paste(ERCC, collapse="|"), gene_names) )

  counts_file_2 <- counts_file_2[!(row.names(counts_file_2) %in% ERCC_genes), ]

  ##############

  assign(data_sets, CreateSeuratObject(counts_file_2) )

}

subsetted_data <- MergeSeurat(get(to_analyze[1]),
                              get(to_analyze[1+1]),
                              do.normalize=FALSE)

remove(list=to_analyze)
remove(counts_file_2)
remove(name_file_2)

#################

mito.genes <- grep(pattern = "^mt-", x = rownames(x = subsetted_data@data),
value = TRUE)

percent.mito <-
  Matrix::colSums(subsetted_data@raw.data[mito.genes, ])/
Matrix::colSums(subsetted_data@raw.data)

subsetted_data <- AddMetaData(object = subsetted_data,
                              metadata = percent.mito, col.name = "percent.mito")

GenePlot(object = subsetted_data, gene1 = "nUMI", gene2 = "percent.mito")
GenePlot(object = subsetted_data, gene1 = "nUMI", gene2 = "nGene")

subsetted_data <- FilterCells(object = subsetted_data,
                    subset.names = c("nGene", "percent.mito"),
                    low.thresholds = c(500, -Inf),
                    high.thresholds = c(4200, 0.03))

#################

subsetted_data <- NormalizeData(subsetted_data, scale.factor=1e6)

###############

s.genes <- cc.genes$s.genes
g2m.genes <- cc.genes$g2m.genes
subsetted_data <- CellCycleScoring(subsetted_data, g2m.genes = g2m.genes,
                                   s.genes = s.genes) #, set.ident = TRUE)

subsetted_data <- ScaleData(subsetted_data, vars.to.regress =
                              c("nUMI", "percent.mito", "S.Score", "G2M.Score"),
                            do.par=TRUE, num.cores=6)
```

```
subsetted_data <- FindVariableGenes(object = subsetted_data,
                   mean.function = ExpMean, dispersion.function = LogVMR)

subsetted_data <- RunPCA(subsetted_data)

subsetted_data <- RunUMAP(subsetted_data, dims.use = 1:20)

# PCElbowPlot(object = qNSC, num.pc = 20)

subsetted_data <- FindClusters(subsetted_data, resolution=0.8, dims.use = 1:20)

##################

gene_to_graph=c("Slc1a3", "Clu", "Id3", "Nr2e1", "Lrig1", "Id1", "Tnfrsf19", "Ascl1",
                "Dlx2", "Stmn2",
                "Egfr", "Aldoc", "Sox2", "Id2", "Sox9", "Sox11", "Gli1", "Gli2",
                "Gli3", "Gfap",
                "Thbs4", "Fezf2", "Sfrp1", "Scml4", "Cd38", "Agt", "Dcx", "Vim",
                "Etnppl", "Rgs6", "Fgfr3", "Aqp4", "S100b","Crym",
                "Prom1", "Vcam1", "Mfge8", "Thrsp", "Cdk6", "Mki67", "Id4", "Hes1",
                "Hes5", "Nes",
                "Aldh1l1", "Hepacam", "Tubb3", "Notch1", "Foxo1", "Ptch1", "Lfng",
                "Notch2",
                "Mcm6", "Mcm2", "Pcna", "Mki67",
                 # reference genes
                "Cldn10", "Slc1a3", "S1pr1", # astrocytes
                "Opalin", "Mog", "Enpp2",  # oligodendrocytes
                "Fcgr3", "Cd53", "Tmem119", # microglia
                "Nrgn", "Pcp4", "Meg3", # neurons
                "Cldn5", "Flt1", "Egfl7", # endothelial cells
                "Enkur", "Foxj1", # ependymal cells
                "Vtn", "Rgs5", # mural cells
                "Pdgfra", "Lhfpl3", # OPC's
                "Gpr17", "Tnr", "Fyn" # COP's
                )

#########

png(file=paste("umap_graph.png", sep=""),bg = "transparent", width=2, height=2,
    units="in", res=300)
fp <-   DimPlot(subsetted_data, reduction="umap", group.by="orig.ident",
                pt.size=.1, cols.use="orange",
                no.legend=TRUE, no.axes=TRUE, do.return = TRUE)
print(
  fp + theme(
    panel.background = element_rect(fill = "transparent"),
    plot.background = element_rect(fill = "transparent", color = NA)
  )
)
dev.off()

png(file=paste("umap_clusters_graph.png", sep=""),bg = "transparent", width=2,
height=2,
    units="in", res=300)
fp <-   DimPlot(subsetted_data, reduction="umap", pt.size=.1, no.legend=TRUE,
no.axes=TRUE,
                do.return = TRUE)
```

```
print(
  fp + theme(
    panel.background = element_rect(fill = "transparent"),
    plot.background = element_rect(fill = "transparent", color = NA)
)
)
dev.off()

for (g in gene_to_graph){
  png(file=paste(g, "_graph.png", sep=""),bg = "transparent", width=2, height=2,
      units="in", res=300)
  fp <- FeaturePlot(subsetted_data, features = g, reduction = "umap",
                    min.cutoff="q10", max.cutoff = "q90", cols.use=c(NA, "red"),
                    pt.size =.1, no.legend=TRUE, no.axes=TRUE, do.return = TRUE)
  print(
    lapply(fp, `+`, theme(
      panel.background = element_rect(fill = "transparent"),
      plot.background = element_rect(fill = "transparent", color = NA)
    ))
  )
  dev.off()
}

png(file=paste("gapdh_graph.png", sep=""),bg = "transparent", width=2, height=2,
    units="in", res=300)
fp <- FeaturePlot(subsetted_data, features = "Gapdh", reduction = "umap",
                  cols.use=c("yellow", "yellow"),
                  pt.size =.1, no.legend=TRUE, no.axes=TRUE, do.return = TRUE)
print(
  lapply(fp, `+`, theme(
    panel.background = element_rect(fill = "transparent"),
    plot.background = element_rect(fill = "transparent", color = NA)
  ))
)
dev.off()


##

six_markers <- FindMarkers(subsetted_data, ident.1=6)
zero_markers <- FindMarkers(subsetted_data, ident.1=0)
eight_markers <- FindMarkers(subsetted_data, ident.1=8)
two_markers <- FindMarkers(subsetted_data, ident.1=2)
four_markers <- FindMarkers(subsetted_data, ident.1=4)
eleven_markers <- FindMarkers(subsetted_data, ident.1=11)
ten_markers <- FindMarkers(subsetted_data, ident.1=10)
eighteen_markers <- FindMarkers(subsetted_data, ident.1=18)

six_vs_zero_markers <- FindMarkers(subsetted_data, ident.1=6, ident.2=0)
zero_vs_six_markers <- FindMarkers(subsetted_data, ident.1=0, ident.2=6)
zero_vs_eight_markers <- FindMarkers(subsetted_data, ident.1=0, ident.2=8)
six_vs_eight_markers <- FindMarkers(subsetted_data, ident.1=6, ident.2=8)

write.table(six_markers,"Astro_markers.txt",sep="\t", col.names=NA)
write.table(zero_markers,"qNSC_markers.txt",sep="\t", col.names=NA)
write.table(eight_markers,"aNSC_markers.txt",sep="\t", col.names=NA)

write.table(six_vs_zero_markers,"Astro_vs_qNSC_markers.txt",sep="\t", col.names=NA)
```

```
write.table(zero_vs_six_markers,"qNSC_vs_Astro_markers.txt",sep="\t", col.names=NA)
write.table(zero_vs_eight_markers,"qNSC_vs_aNSC_markers.txt",sep="\t", col.names=NA)
write.table(six_vs_eight_markers,"Astro_vs_aNSC_markers.txt",sep="\t", col.names=NA)

write.table(two_markers,"TAC_markers.txt",sep="\t", col.names=NA)
write.table(four_markers,"NB_markers.txt",sep="\t", col.names=NA)
write.table(eleven_markers,"ependymal_cell_markers.txt",sep="\t", col.names=NA)
write.table(ten_markers,"ten_markers.txt",sep="\t", col.names=NA)
write.table(eighteen_markers,"eighteen_markers.txt",sep="\t", col.names=NA)

###########

Astro <- row.names(subset(subsetted_data@meta.data, subset=(res.0.8==6)))
qNSC <- row.names(subset(subsetted_data@meta.data, subset=(res.0.8==0)))
aNSC <- row.names(subset(subsetted_data@meta.data, subset=(res.0.8==8)))
TAC <- row.names(subset(subsetted_data@meta.data, subset=(res.0.8==2)))
NB <- row.names(subset(subsetted_data@meta.data, subset=(res.0.8==4)))
EpC <- row.names(subset(subsetted_data@meta.data, subset=(res.0.8==11)))

#############

Astro_cells_data <- SubsetData(subsetted_data, cells.use=as.character(Astro))

Astro_genes <- data.frame(mean.exp = rowMeans(expm1(x = Astro_cells_data@data)))
Astro_genes <- subset(Astro_genes, subset=(mean.exp>0))
Astro_genes <- Astro_genes[order(Astro_genes$mean.exp, decreasing = TRUE),, drop =
FALSE]

write.table(Astro_genes,"Astro_expressed_genes.txt",sep="\t", col.names=NA)

#############

qNSC_cells_data <- SubsetData(subsetted_data, cells.use=as.character(qNSC))

qNSC_genes <- data.frame(mean.exp = rowMeans(expm1(x = qNSC_cells_data@data)))
qNSC_genes <- subset(qNSC_genes, subset=(mean.exp>0))
qNSC_genes <- qNSC_genes[order(qNSC_genes$mean.exp, decreasing = TRUE),, drop = FALSE]

write.table(qNSC_genes,"qNSC_expressed_genes.txt",sep="\t", col.names=NA)

#############

aNSC_cells_data <- SubsetData(subsetted_data, cells.use=as.character(aNSC))

aNSC_genes <- data.frame(mean.exp = rowMeans(expm1(x = aNSC_cells_data@data)))
aNSC_genes <- subset(aNSC_genes, subset=(mean.exp>0))
aNSC_genes <- aNSC_genes[order(aNSC_genes$mean.exp, decreasing = TRUE),, drop = FALSE]

write.table(aNSC_genes,"aNSC_expressed_genes.txt",sep="\t", col.names=NA)

#############

TAC_cells_data <- SubsetData(subsetted_data, cells.use=as.character(TAC))

TAC_genes <- data.frame(mean.exp = rowMeans(expm1(x = TAC_cells_data@data)))
TAC_genes <- subset(TAC_genes, subset=(mean.exp>0))
TAC_genes <- TAC_genes[order(TAC_genes$mean.exp, decreasing = TRUE),, drop = FALSE]
```

```
write.table(TAC_genes,"TAC_expressed_genes.txt",sep="\t", col.names=NA)

#############

NB_cells_data <- SubsetData(subsetted_data, cells.use=as.character(NB))

NB_genes <- data.frame(mean.exp = rowMeans(expm1(x = NB_cells_data@data)))
NB_genes <- subset(NB_genes, subset=(mean.exp>0))
NB_genes <- NB_genes[order(NB_genes$mean.exp, decreasing = TRUE),, drop = FALSE]

write.table(NB_genes,"NB_expressed_genes.txt",sep="\t", col.names=NA)

####################

ependymal_cells_data <- SubsetData(subsetted_data, cells.use=as.character(EpC))

EpC_genes <- data.frame(mean.exp = rowMeans(expm1(x = ependymal_cells_data@data)))
EpC_genes <- subset(EpC_genes, subset=(mean.exp>0))
EpC_genes <- EpC_genes[order(EpC_genes$mean.exp, decreasing = TRUE),, drop = FALSE]

write.table(EpC_genes,"ependymal_cells_expressed_genes.txt",sep="\t", col.names=NA)

####################

  print("Total number of processed cells")
  total_processed_cells<-
    rbind(
      data.frame(name=Astro),
      data.frame(name=qNSC),
      data.frame(name=aNSC),
      data.frame(name=TAC),
      data.frame(name=NB)
    )

  print(
  nrow(total_processed_cells)
  )

# quick check of the overlap between a few cell types

Astro_qNSC<-list("Astro"=unlist(Astro),"qNSC"=unlist(qNSC))
venn(Astro_qNSC)

qNSC_aNSC<-list("qNSC"=unlist(qNSC),"aNSC"=unlist(aNSC))
venn(qNSC_aNSC)

aNSC_TAC<-list("aNSC"=unlist(aNSC),"TAC"=unlist(TAC))
venn(aNSC_TAC)

print("qNSC and aNSC")
print(
  length(intersect(qNSC, aNSC))
)

print("qNSC and TAC")
print(
  length(intersect(qNSC, TAC))
)
```

```
print("qNSC and NB")
print(
  length(intersect(qNSC, NB))
)

###############

data_to_graph <- data.frame()

for (g in gene_to_graph){

  data_temp <- data.frame()
  if (length( Astro ) > 0)
  { data_temp <- data.frame(cell_type="Astro", gene_name=g,
            expression_level=as.numeric(unlist(FetchData(object=subsetted_data,
                          cells.use=Astro, vars.all=g))))
  data_to_graph <- rbind(data_to_graph, data_temp) } else
  {
    data_temp <- data.frame(cell_type="Astro", gene_name=g, expression_level=NA)
    data_to_graph <- rbind(data_to_graph, data_temp) }

  data_temp <- data.frame()
  if (length( qNSC ) > 0)
  { data_temp <- data.frame(cell_type="qNSC", gene_name=g,
            expression_level=as.numeric(unlist(FetchData(object=subsetted_data,
                          cells.use=qNSC, vars.all=g))))
  data_to_graph <- rbind(data_to_graph, data_temp) } else
  {
    data_temp <- data.frame(cell_type="qNSC", gene_name=g, expression_level=NA)
    data_to_graph <- rbind(data_to_graph, data_temp) }

  data_temp <- data.frame()
  if (length( aNSC ) > 0){
    data_temp <- data.frame(cell_type="aNSC", gene_name=g,
            expression_level=as.numeric(unlist(FetchData(object=subsetted_data,
                          cells.use=aNSC, vars.all=g))))
    data_to_graph <- rbind(data_to_graph, data_temp)} else
    {
      data_temp <- data.frame(cell_type="aNSC", gene_name=g, expression_level=NA)
      data_to_graph <- rbind(data_to_graph, data_temp)}

  data_temp <- data.frame()
  if (length( TAC ) > 0){
    data_temp <- data.frame(cell_type="TAC", gene_name=g,
            expression_level=as.numeric(unlist(FetchData(object=subsetted_data,
                          cells.use=TAC, vars.all=g))))
    data_to_graph <- rbind(data_to_graph, data_temp)} else
    {
      data_temp <- data.frame(cell_type="TAC", gene_name=g, expression_level=NA)
      data_to_graph <- rbind(data_to_graph, data_temp)}

  data_temp <- data.frame()
  if (length( NB ) > 0){
    data_temp <- data.frame(cell_type="NB", gene_name=g,
            expression_level=as.numeric(unlist(FetchData(object=subsetted_data,
                          cells.use=NB, vars.all=g))))
    data_to_graph <- rbind(data_to_graph, data_temp)} else
```

```
    {
      data_temp <- data.frame(cell_type="NB", gene_name=g, expression_level=NA)
      data_to_graph <- rbind(data_to_graph, data_temp)}
}

# exclude zeros for visualization of the mean in expressing cells

data_to_graph_2 <- data.frame()
data_to_graph_2 <- subset(data_to_graph, subset=(expression_level > 0))

############

data_to_graph$cell_type <- factor(data_to_graph$cell_type,
                                  levels=c("Astro", "qNSC", "aNSC", "TAC", "NB"))

data_to_graph_2$cell_type <- factor(data_to_graph_2$cell_type,
                                    levels=c("Astro", "qNSC", "aNSC", "TAC", "NB"))

###############

for (graphs in gene_to_graph){

  pdf(file=paste(graphs, ".pdf", sep=""), width=5, height=4, useDingbats=FALSE)

  print(
    ggplot() +

      theme(
        panel.background = element_rect(fill = "white"),
        plot.title = element_text(hjust = 0.5),
        legend.position="none",
        axis.line = element_line(color="black", size=.5),
        axis.title.x = element_text(size=14),
        axis.text.x = element_text(size=14, angle=25, hjust=1, color="black"),
        axis.title.y = element_text(size=14),
        axis.text.y = element_text(size=14, color="black"),
        strip.text.x = element_text(size = 14),
        axis.ticks = element_line(color="black") ) +

      scale_x_discrete(name="Cell types") +

      scale_y_continuous(expand = c(0,0), limits=c(0,
               (max(
               subset(data_to_graph,
               subset=(complete.cases(data_to_graph) & gene_name==graphs),
                       select=expression_level) )*1.1 ) ),
                         name="Expression level") +

      # for some reason, the x-axis order is rearranged, easy fix
      geom_point(data=subset(data_to_graph, subset=(gene_name==graphs)),
                 aes(x=cell_type, y=expression_level),
                 stat="identity", position_jitter(height = 0, width = 0.15),
                 shape=21, color="black", fill = "magenta", size=0, stroke=0) +

      geom_violin(data=subset(data_to_graph, subset=(gene_name==graphs)),
                 aes(x=cell_type, y=expression_level)) +

      geom_point(data=subset(data_to_graph, subset=(gene_name==graphs)),
```

```
                 aes(x=cell_type, y=expression_level),
                 stat="identity", position_jitter(height = 0, width = 0.15),
                 shape=21, color="black", fill = "magenta", size=3, stroke=.1)

  )

  dev.off()

}

dev.off()

######################

# concatenate the names of all subsetted cells and export to a file
# for additional analyses in the downstream scripts

all_cell_names <- rbind(data.frame(name=Astro), data.frame(name=qNSC),
                        data.frame(name=aNSC), data.frame(name=TAC),
                        data.frame(name=NB) )

write.table(all_cell_names,"all_cell_names.txt",sep="\t",row.names=FALSE)

print("Number of all cells sorted in silico")

print(nrow(all_cell_names))

#######################

all_cell_names <- read.table("all_cell_names.txt", header=TRUE, sep="\t")

all_cells_data <- SubsetData(subsetted_data,
                             cells.use=as.character(all_cell_names$name))

# check whether the SubsetData worked correctly
# compare to the previous list

subsetted_names <- data.frame(name=row.names(all_cells_data@meta.data))

print("Number of cells that are different")

print(
  length(setdiff(as.character(all_cell_names$name),
                 as.character(subsetted_names$name)))
)

# subset worked correctly

##################

all_cells_monocle_data <- importCDS(all_cells_data, import_all=TRUE)

all_cells_monocle_data <- estimateSizeFactors(all_cells_monocle_data)

all_cells_monocle_data <- estimateDispersions(all_cells_monocle_data)

disp_table <- dispersionTable(all_cells_monocle_data)
```

```
all_cells_monocle_data  <- detectGenes(all_cells_monocle_data ,
                                       min_expr = 0.1)

expressed_genes <-  data.frame(name=
                     row.names(subset(fData(all_cells_monocle_data),
                            subset=(num_cells_expressed >= 100))))
#100 for 29k, 10 for 13k

##############

# semi-supervised pseudotemporal ordering

cth <- newCellTypeHierarchy()

# cth <- addCellType(cth,
#                    "Astrocytes",
#                    classify_func = function(x) { x["Slc1a3",] >= 3 }) #3
#
# Stem_id <- c("Prom1")
#
# cth <- addCellType(cth,
#                    "GFAP+ stem cell astrocytes",
#                    classify_func = function(x) { x[Stem_id,] >= 3 &
# x["Gfap",] >=3 }, #3
#                    parent_cell_type_name="Astrocytes")
#
# cth <- addCellType(cth,
#                    "GFAP- stem cell astrocytes",
#                    classify_func = function(x) { x[Stem_id,] >= 3 &
# x["Gfap",] <1 }, #3
#                    parent_cell_type_name="Astrocytes")
#
# cth <- addCellType(cth,
#                    "GFAP+ non-stem cell astrocytes",
#                    classify_func = function(x) { x[Stem_id,] <1 &
# x["Gfap",] >=3 }, #3
#                    parent_cell_type_name="Astrocytes")
#
# cth <- addCellType(cth,
#                    "GFAP- non-stem cell astrocytes",
#                    classify_func = function(x) { x[Stem_id,] <1 &
# x["Gfap",] <1 }, #3
#                    parent_cell_type_name="Astrocytes")

cth <- addCellType(cth,
                   "TAC's",
                   classify_func = function(x) { x["Mki67",] >= 3 }) #3

cth <- addCellType(cth,
                   "Nb's",
                   classify_func = function(x) { x["Stmn2",] >= 3 }) #3

# simplest, let the algorithm do the ordering without specifying
# any stem cell marker, generates same result as including the above criteria

############

all_cells_monocle_data <- classifyCells(all_cells_monocle_data, cth)
```

```
marker_diff <- markerDiffTable(
  all_cells_monocle_data[as.character(expressed_genes$name)],
   cth, cores = 6)

semisup_clustering_genes <-
  row.names(marker_diff)[order(marker_diff$qval)][1:1000]

all_cells_monocle_data <- setOrderingFilter(all_cells_monocle_data,
                                            semisup_clustering_genes)

all_cells_monocle_data <- reduceDimension(all_cells_monocle_data,
                                       max_components = 2,
                                       reduction_method = c("DDRTree") )

all_cells_monocle_data <- orderCells(all_cells_monocle_data)

##############

# sometimes necessary
all_cells_monocle_data <- orderCells(all_cells_monocle_data, root_state=3)

dev.off()

selected_genes <- c("Slc1a3", "Ascl1", "Dcx", "Meg3", "Mki67")

png(file=paste("selected_genes_in_pseudotime.png", sep=""),
    bg = "transparent", width=5, height=4, units="in", res=300)
print(
  plot_genes_in_pseudotime(all_cells_monocle_data[selected_genes,],
                                 ncol = 1)
  +  theme(legend.position = "none",
           axis.ticks = element_line(colour = "black", size = .2),
           axis.text.x = element_text(size = 6),
           axis.text.y = element_text(size = 6)
  )
)
dev.off()


dev.off()

png(file=paste("pseudotime.png", sep=""), bg="transparent", width=5, height=4,
    units="in", res=300)
print(
  plot_cell_trajectory(all_cells_monocle_data, cell_size=0.1,
                       color_by = "Pseudotime",
                       show_tree=FALSE, show_branch_points = FALSE) +
  theme(legend.position = "none",
        strip.text.x = element_text(size=0)  )
  )
dev.off()

#################

gene_to_graph=c("Slc1a3", "Clu", "Nr2e1", "Lrig1", "Ascl1", "Dlx2",
                "Egfr", "Sox2", "Id2", "Sox9", "Sox11",
                "Thbs4", "Agt", "Dcx", "Etnppl",
```

```
                 "Vcam1", "Mfge8", "Cdk6", "Mki67", "Id4", "Hes1", "Hes5",
                "Tubb3", "Lfng")

dev.off()

for (g in gene_to_graph){
  pdf(file=paste(g, "_pseudotime.pdf", sep=""), width=40, height=32,
      useDingbats=FALSE)
  print(
    plot_cell_trajectory(all_cells_monocle_data, markers = g,
use_color_gradient=FALSE,
                         markers_linear=TRUE, cell_size=1,
                         show_tree=FALSE, show_branch_points = FALSE) +
      theme(legend.position = "none",
            strip.text.x = element_text(size=0) )
  )
  dev.off()
}

dev.off()

pdf(file=paste("branched_pseudotime.pdf", sep=""), width=8, height=10,
    useDingbats=FALSE)
plot_genes_branched_heatmap(all_cells_monocle_data[gene_to_graph,],
                            branch_point = 1,
                            cores = 6,
                            use_gene_short_name = T,
                            show_rownames = T)
dev.off()


dev.off()

pdf(file=paste("pseudotime_heatmap.pdf", sep=""), width=8, height=10,
    useDingbats=FALSE)
plot_pseudotime_heatmap(all_cells_monocle_data[gene_to_graph,],
                        cores = 6,
                        show_rownames = T)
dev.off()

#################################
#################################
#################################
```
